# Isolation and Genomics of *Futiania mangrovii* gen. nov., sp. nov., a Rare and Metabolically Versatile Member in the Class *Alphaproteobacteria*

**DOI:** 10.1128/spectrum.04110-22

**Published:** 2022-12-21

**Authors:** Lirui Liu, Wen-Cong Huang, Jie Pan, Jiayi Li, Yuhan Huang, Dayu Zou, Huan Du, Yang Liu, Meng Li

**Affiliations:** a Archaeal Biology Center, Institute for Advanced Study, Shenzhen University, Shenzhen, China; b Key Laboratory of Optoelectronic Devices and Systems, College of Physics and Optoelectronic Engineering, Shenzhen University, Shenzhen, China; c Shenzhen Key Laboratory of Marine Microbiome Engineering, Institute for Advanced Study, Shenzhen University, Shenzhen, China; d Department of Marine Microbiology and Biogeochemistry, NIOZ, Royal Netherlands Institute for Sea Research, Den Burg, the Netherlands; e Institute for Biodiversity and Ecosystem Dynamics (IBED), University of Amsterdam, Amsterdam, the Netherlands; f Shenzhen Xbiome Biotech Co. Ltd., Shenzhen, China; Nanjing Institute of Geography and Limnology, Chinese Academy of Sciences

**Keywords:** novel alphaproteobacterium, mangrove sediment isolate, microbial adaptation

## Abstract

Mangrove microorganisms are a major part of the coastal ecosystem and are directly associated with nutrient cycling. Despite their ecological significance, the collection of culturable mangrove microbes is limited due to difficulties in isolation and cultivation. Here, we report the isolation and genome sequence of strain FT118^T^, the first cultured representative of a previously uncultivated order UBA8317 within *Alphaproteobacteria*, based on the combined results of 16S rRNA gene similarity, phylogenomic, and average amino acid identity analyses. We propose *Futianiales* ord. nov. and *Futianiaceae* fam. nov. with *Futiania* as the type genus, and FT118^T^ represents the type species with the name *Futiania mangrovii* gen. nov, sp. nov. The 16S rRNA gene sequence comparison reveals that this novel order is a rare member but has a ubiquitous distribution across various habitats worldwide, which is corroborated by the experimental confirmation that this isolate can physiologically adapt to a wide range of oxygen levels, temperatures, pH and salinity levels. Biochemical characterization, genomic annotation, and metatranscriptomic analysis of FT118^T^ demonstrate that it is metabolically versatile and active *in situ*. Genomic analysis reveals adaptive features of *Futianiales* to fluctuating mangrove environments, including the presence of high- and low-affinity terminal oxidases, N-type ATPase, and the genomic capability of producing various compatible solutes and polyhydroxybutyrate, which possibly allow for the persistence of this novel order across various habitats. Collectively, these results expand the current culture collection of mangrove microorganisms, providing genomic insights of how this novel taxon adapts to fluctuating environments and the culture reference to unravel possible microbe-environment interactions.

**IMPORTANCE** The rare biosphere constitutes an essential part of the microbial community and may drive nutrient cycling and other geochemical processes. However, the difficulty in microbial isolation and cultivation has hampered our understanding of the physiology and ecology of uncultured rare lineages. In this study, we successfully isolated a novel alphaproteobacterium, designated as FT118^T^, and performed a combination of phenotypic, phylogenetic, and phylogenomic analyses, confirming that this isolate represents the first cultured member of a previously uncultivated order UBA8317 within *Alphaproteobacteria*. It is a rare species with a ubiquitous distribution across different habitats. Genomic and metatranscriptomic analyses demonstrate that it is metabolically versatile and active *in situ*, suggesting its potential role in nutrient cycling despite being scarce. This work not only expands the current phylogeny of isolated *Alphaproteobacteria* but also provides genomic and culture reference to unravel microbial adaptation strategies in mangrove sediments and possible microbe-environment interactions.

## INTRODUCTION

Ubiquitously found in every possible ecosystem, microorganisms are the most phylogenetically and metabolically diverse life form on Earth. Due to recent advances in sequencing technology and computational methods, previously known microbial diversity has been significantly expanded by metagenome-assembled genomes (MAGs) and single-amplified genomes (SAGs). Notably, Parks et al. successfully recovered nearly 8,000 MAGs from more than 1,500 metagenomes, and these uncultivated bacteria and archaea (UBA) genomes contributed to a substantially diversified genomic representation of prokaryotic lineages, including 20 novel bacterial and archaeal phyla (1). Later, projects at even larger scales provided access to comprehensive genomic catalogs that cover all types of environments and unparalleled insights into microbial ecology and evolution (2, 3). Yet extensive efforts in deep metagenomic sequencing cannot replace cultivation-dependent approaches in microbiology (4). Although continuous culturing efforts have led to annual increases of over 1,000 novel bacterial species published in the past few years (5), the number of microbial strains maintained in pure cultures or cocultures only represents a minimal fraction of the global microbial diversity existing on Earth. Strikingly, among at least 60 major prokaryotic branches on the tree of life, half of them lack isolated representatives, which are referred to as microbial dark matter (6, 7). Current documentation of 89,545 strains of 16,504 species in the BacDive database (as of March 2022) has a skewed distribution, as almost 89% of microbial isolates belong to four bacterial phyla: *Actinomycetota*, *Pseudomonadota*, *Bacillota*, and *Bacteroidota* (8). The large proportion of uncultured microbiota hampers our understanding of the physiology and cell biology of these uncultured lineages as well as their ecological roles in global nutrient cycles.

*Alphaproteobacteria* is often found as one of the most metabolically active and numerically abundant taxa across various habitats, including marine, freshwater, soil, lichens, and host-related habitats. Its members show a remarkable degree of genome plasticity associated with free-living, intracellular, and facultative lifestyles (9). According to the Genome Taxonomy Database (GTDB R202), *Alphaproteobacteria* consists of 97 distinct orders, while only 17 orders are included in List of Prokaryotic names with Standing in Nomenclature (LPSN) and contain isolated representatives. At present, there are nearly 4,000 microbial isolates described within the class *Alphaproteobacteria*, most of which fall into the orders *Hyphomicrobiales*, *Rhodobacterales*, *Sphingomonadales*, and *Rhodospirillales*, whereas several newly established orders contain only one cultured representative, such as *Emcibacterales*, *Magnetococcales*, *Minwuiales*, and *Rhodothalassiales* (8). In GTDB, one of the uncultivated alphaproteobacterial orders UBA8317 has four MAGs obtained from global marine metagenomes, including three MAGs from marine water samples of the Mediterranean Sea (10–12) and one MAG from unknown geographic location (1). So far, no isolation or description has been reported regarding this group.

Showing immense ecological and economic significance, mangrove ecosystems function as a hot spot for energy flow and nutrient cycling. Diverse mangrove microbiota plays a major role in sustaining the great biodiversity and high productivity of mangrove ecosystems (13). Many factors contribute to substantial differences in the mangrove microbial community structure, including seasonal changes (14), mangrove species (15), environmental factors, such as pH, salinity, total carbon, and total nitrogen (16), and human activities (17). Specifically, research focusing on mangrove ecosystems has suggested that mean annual precipitation and total nitrogen have a significant negative effect on *Alphaproteobacteria*, while *Betaproteobacteria* and Deltaproteobacteria abundances are negatively correlated with pH (16). In comparison, the abundance of *Pseudomonadota* as a whole did not show strong correlation with tested environmental variables in estuarine wetland soil samples (18).

Located in Shenzhen, Guangdong Province, Futian National Nature Reserve (FNNR) is the only mangrove forest in urban area of China, and its major mangrove species include *Aegiceras corniculatum*, *Acanthus ilicifolius*, *Bruguiera gymnorhiza*, and *Kandelia candel* (19). A previous study has revealed that *Pseudomonadota* is one of the most abundant bacterial lineages in the mangrove sediments at FNNR, among which *Alphaproteobacteria* accounts for nearly one-third of the *Pseudomonadota* abundance (19), making FNNR an ideal isolation source of uncultivated *Alphaproteobacteria*. Here, we report the isolation of the first cultured representative of UBA8317, strain FT118^T^, and its genome sequence. We also provide a description of its physiological, biochemical, and chemotaxonomic features and the ecological distribution of this novel order. Our results demonstrate its adaptability to a wide range of environmental variables and reveal its genomic features that allow its adaptation to fluctuating mangrove environments. This expands our current culture collections of mangrove microorganisms and provides a reference in future ecological and taxonomical studies.

## RESULTS AND DISCUSSION

### Phenotypic characterization.

**FIG 1:**
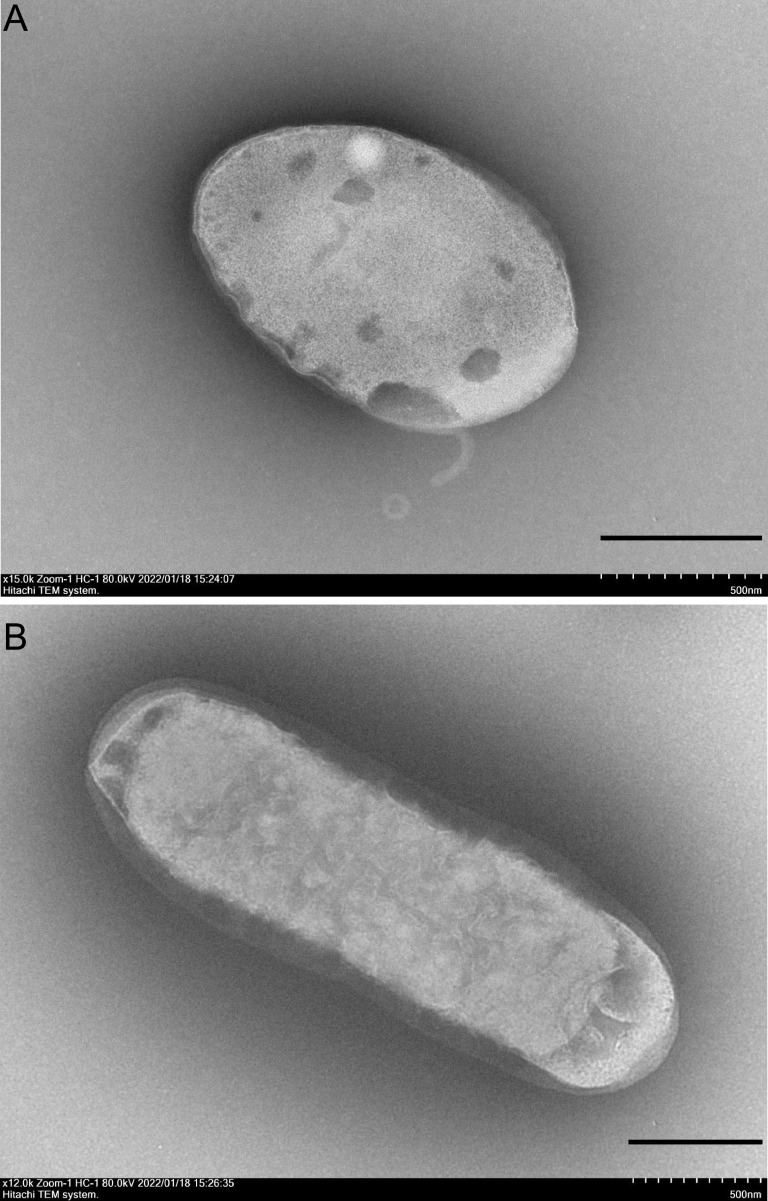
Transmission electron microscope images of strain FT118^T^. Cells were grown in 2216 broth at 37°C to exponential phase; scale bars, 500 nm.

Strain FT118^T^ was isolated from the 0 to 10 cm layer of the collected mangrove sediment by directly plating serial dilutions on 2216 agar. The colonies were white, circular, raised, and smooth with entire edges on 2216 agar. Cells of strain FT118^T^ were Gram negative and motile. During exponential phase, ovoid to rod-shaped cells could be observed with sizes of approximately 1.2 to 4 μm long and 0.5 to 0.9 μm wide (Fig. 1). Strain FT118^T^ could grow under anaerobic conditions, but the growth was rather limited compared to that under aerobic conditions. Strain FT118^T^ could grow within 20 to 45°C, and its optimal temperature was 30 to 40°C. Additionally, strain FT118^T^ grew within a pH range of 6 to 10, with an optimal pH of 6 to 7, and was able to grow within a NaCl concentration range of 0 to 13% (wt/vol); growth optimum was at 2% (Fig. S1 in the supplemental material).

Strain FT118^T^ was catalase positive and oxidase positive. It did not hydrolyze casein, starch, or cellulose. In the API 20NE test, strain FT118^T^ was positive in the reduction of nitrate to nitrite, gelatin hydrolysis, and β-galactosidase activity. In the API ZYM test, strain FT118^T^ had positive reactions in alkaline phosphatase, esterase (C4), esterase lipase (C8), lipase (C14), leucine arylamidase, cysteine arylamidase, and naphtol-AS-BI-phosphohydrolase. Strain FT118^T^ was weakly positive in acid phosphatase activity. In the API 50CH test, strain FT118^T^ showed only weak acid production from potassium 5-ketogluconate. The result of the Biolog GENIII test revealed that *N*-acetylneuraminic acid, myo-inositol, d-fructose-6-phosphate (d-fructose-6P), and glucuronamide could be used as the sole carbon source. In addition, use of some simple sugars, such as fructose, fucose, and rhamnose, was weakly positive. These results suggest that strain FT118^T^ is an organoheterotroph; some of its characteristics are listed in Table 1 and are compared to representative species of *Alphaproteobacteria* (20–24). The complete results of the API ZYM, API 20NE, and Biolog GENIII tests are summarized in Table S1.

**TABLE 1:** Characteristics of FT118^T^ and representative species of related orders in *Alphaproteobacteria*

Characteristics	FT118^T^^*a*^	Rhodobacter capsulatus GDMCC 1.168^T^^*a*^^,^^*b*^	Caulobacter vibrioides DSM 4738^T^^*a*^^,^^*c*^	Parvularcula bermudensis HTCC2503^T^^*a*^^,^^*d*^	Micropepsis pineolensis JCM 30711^T^^*a*^^,^^*e*^
Order	*Futianiales* ord. nov.	*Rhodobacterales*	*Caulobacterales*	*Parvularculales*	*Micropepsales*
Cell morphology	Ovoid to rods	Ovoid to rods	Rods	Short rods	Slightly curved rods
O_2_ requirement	Facultatively aerobic	Anaerobic	Aerobic	Aerobic	Anaerobic
Mobility	+	+	+	+	−
Flagella	−	+	+	+	ND
Temperature range (optimum °C)	20–45 (30–40)	15–40 (30–35)	ND–43 (30)	10–37 (30)	15–35 (35)
pH range (optimum)	6–10 (6–7)	6–8.3 (7–7.5)	5.5–7.5 (6.0–6.5)	6–9 (8)	5.0–6.8 (5.6)
NaCl tolerance (optimum [wt/vol])	0–13% (2%)	0–2% (0–0.6%)	0–0.5% (ND)	0.75–20% (3%)	0–0.58% (0–0.29%)
DNA G+C content (%)	68.5	68.8	64–65	60.8	61.9
Catalase	+	ND	+	−	−
Oxidase	+	ND	w	+	−
Major fatty acids (>5% of total fatty acids)	C18:1 ω7*c* (54.06%), C19:0 cyclo ω8*c* (16.1%), C16:0 (7.62%)	C18:1 ω7*c* (75.98%), C16:1 ω7*c*/16:1 ω6*c* (8.43%), C18:0 iso (7.59%)	C18:1 ω7*c* (35.5%), C16:0 (16.7%), iso-C17:0 (11.2%), iso-C15:0 (8.9%), 11-methyl C18:1 ω7*c* (6.2%)	C18:1 ω7*c* (73.3%), C16:0 (8.6%), C18:1 ω9*c* (6%), C12:0 (5.2%)	C18:1 ω7*c* (36.7%), C19:0 cyclo ω8*c* (22.0%), C14:0 (17.0%), C16:0 (13.2%)
Polar lipids	DPG, PE, PME, AL, APL, PL	PE, PG, PC, AGL, AL	PG	ND	ND
Quinone	Ubiquinone Q10	Ubiquinone Q10	Ubiquinone Q10	ND	ND

a +, positive; −, negative; w, weakly positive; ND, no data available; DPG, diphosphatidylglycerol; PE, phosphatidylethanolamine; PME, phosphatidylmethylethanolamine; PG, phosphatidylglycerol; PC, phosphatidylcholine; AL, unidentified aminolipid; AGL, unknown aminoglycolipid; APL, unidentified aminophospholipid; PL, unidentified phospholipid.

b Data for Rhodobacter capsulatus GDMCC 1.168^T^ is obtained from reference 95 and this study.

c Data for Caulobacter vibrioides DSM 4738^T^ is obtained from references 96, 97.

d Data for Parvularcula bermudensis HTCC 2503^T^ is obtained from reference 20.

e Data for Micropepsis pineolensis JCM 30711^T^ is obtained from reference 21.

The major fatty acid (≥5%) compositions of strain FT118^T^ were C18:1 ω7*c* (54.06%), C19:0 cyclo ω8*c* (16.1%), and C16:0 (7.62%), among which C18:1 ω7*c* was also the predominant cellular fatty acid component in other representative species of closely related orders with various percentages. The rest of the cellular fatty acid profiles of these representative species showed remarkable differences in composition and abundance (Table 1; Table S2). The polar lipid profile of strain FT118^T^ contained diphosphatidylglycerol (DPG), phosphatidylethanolamine (PE), phosphatidylmethylethanolamine (PME), an unidentified aminolipid (AL), an unidentified aminophospholipid (APL), and an unidentified phospholipid (PL), which substantially differed from that of Rhodobacter capsulatus GDMCC 1.168^T^ and Caulobacter vibrioides DSM 4738^T^ (Table 1; Fig. S2). The isoprenoid quinone of strain FT118^T^ was ubiquinone Q10 (data not shown), a feature of most members within *Alphaproteobacteria* (25).

### Genome statistics and taxonomy.

The genome size of strain FT118^T^ was 3,397,356 bp with 6 contigs (≥1,000 bp). The largest contig was 1,448,022 bp and *N*_50_ was 1,219,768 bp. The genome was estimated to be 99.57% complete and had 0.22% contamination. The DNA G+C content calculated from the genome sequence was 68.5%. There were 3,190 putative protein-coding genes, 46 tRNA genes, and 3 rRNA genes, including 1 copy of 5S, 16S, and 23S rRNA genes, respectively. No CRISPR-Cas system was detected in the genome.

**FIG 2:**
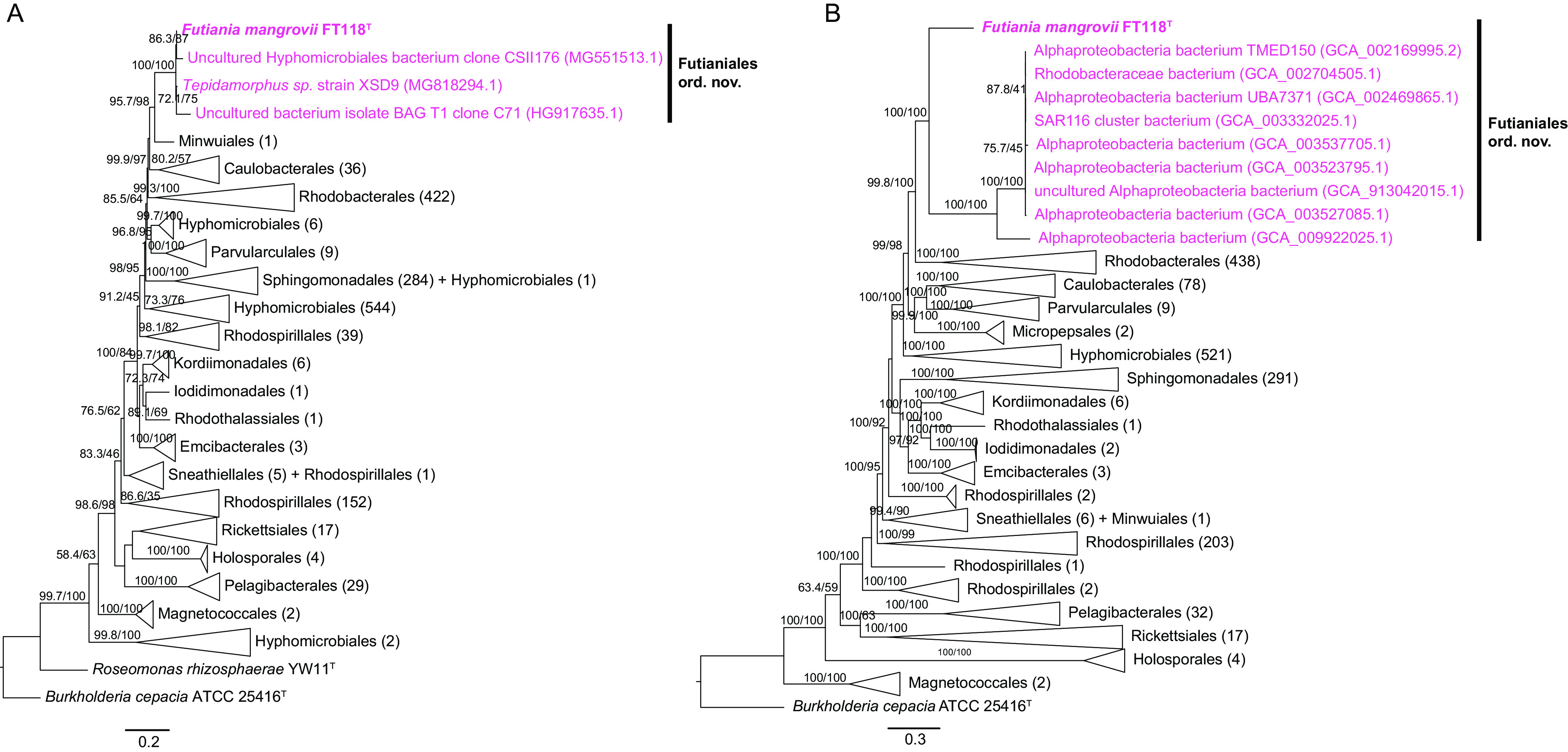
Phylogenetic placement of strain FT118^T^ within the class *Alphaproteobacteria*. (A) A 16S rRNA gene phylogeny reconstructed using nearly full-length 16S rRNA gene sequences of all type strains in *Alphaproteobacteria* (*n* = 1,567) and similar sequences identified in the Silva database (*n* = 3); scale bar, 0.2 nucleotide substitutions per position. (B) Genome-based phylogeny reconstructed using all type strains in *Alphaproteobacteria* (*n* = 1,621) and an additional 9 genomes identified from GenBank that fall into the same order with FT118^T^. A concatenated alignment of 120 conserved protein sequences from GTDB-Tk was used to obtain the phylogeny; scale bar, 0.3 nucleotide substitutions per position. For both trees, branches were collapsed to display the order level. Numbers in the parentheses represent the number of sequences included in each clade. Trees were rooted using Burkholderia cepacia ATCC 25416^T^. Bootstrap values lower than 50 are not shown.

BLAST results of the 16S rRNA gene sequence of FT118^T^ against the Ezbiocloud database revealed that it shared the highest sequence identity with Tepidamorphus gemmatus CB-27A^T^ (92.15%). We also searched the Silva database (138.1 release) and found three 16S rRNA gene sequences that were highly similar to FT118^T^, which belonged to *Tepidamorphus* sp. XSD9 (99.3%), uncultured *Hyphomicrobiales* bacterium clone CSII176 (97.8%), and uncultured bacterium isolate BAG T1 clone C71 (97%). All other hits shared a similarity not higher than 92%. According to the 16S rRNA gene phylogeny, strain FT118^T^ formed a highly supported cluster with these three 16S rRNA gene sequences, and this cluster was the most closely related to *Minwuia thermotolerans* BY3-13^T^ from the order *Minwuiales* (Fig. 2A). However, genome-based classification via GTDB-Tk suggests that this strain belongs to a previously uncultivated order UBA8317 within *Alphaproteobacteria*. Therefore, we searched all alphaproteobacterial genomes (*n* = 23,951, as of October 2021) deposited at the NCBI GenBank database and obtained 9 genomes that also belonged to the order UBA8317. These are all MAGs or SAGs with various genome sizes. CheckM estimated that they were at least 60% complete and had less than 10% contamination (Table S3). Thus, we included these 9 genomes in the phylogenetic analysis together with all the genomes of type strains in *Alphaproteobacteria* (*n* = 1,621, as of June 2021). The genome phylogeny based on 120 concatenated conserved proteins used by GTDB showed that strain FT118^T^ formed a monophyletic clade with all these UBA8317 genomes (UFBOOT: 100; SH-aLRT: 100) and was basal to them in the phylogeny. The lineage containing strain FT118^T^ and other UBA8317 genomes was clearly separated from all other established orders within *Alphaproteobacteria* with strong support (UFBOOT: 99.8; SH-aLRT: 100) (Fig. 2B). The lineage was a sister clade to the order *Rhodobacterales*, which encompasses diverse photoautotrophic and photoheterotrophic bacteria and is believed to be ecologically important (26). In general, the clustering pattern of major alphaproteobacterial lineages in our genome phylogeny is in agreement with previously reported phylogenies based on different protein concatenations (9, 27). Like our observation in *Alphaproteobacteria*, the topological difference between the 16S rRNA gene phylogeny and the genome phylogeny has been widely documented in diverse taxa, for example, *Intrasporangiaceae* and *Mycobacteriaceae* from the phylum *Actinomycetota* (28), Pseudomonas (29), and *Aeromonas* (30) from the phylum *Pseudomonadota* as well as the class *Negativicutes* in the phylum *Bacillota* (31). Although the 16S rRNA gene is one of the most common phylogenetic markers, sometimes it does not provide enough resolution to determine the precise phylogenetic relationship at higher taxonomic ranks (32). Moreover, subtle nucleotide variations between multiple rRNA operons in one genome and possible horizontally transferred 16S rRNA genes may give rise to distorted phylogenetic placements (33–35). In comparison, whole genome-based phylogeny inferred from multiple marker genes provides more phylogenetic information and is generally considered more robust for refined phylogenetic relationships (31).

Besides genome-based phylogeny, we also performed pairwise average nucleotide identity (ANI) and average amino acid identity (AAI) comparisons between strain FT118^T^ and the genomes of alphaproteobacterial type strains. At the nucleotide sequence level, whole-genome comparison by ANI indicated that strain FT118^T^ was divergent from all other established orders of *Alphaproteobacteria*. Strain FT118^T^ shared the highest ANI value with a *Rhodobacterales* bacterium *Polymorphum gilvum* SL003B-26A1 (77.7%), while the ANI values between strain FT118^T^ and many other representative genomes were below 75% (data not shown), including UBA8317 genomes, which was too low for reliable comparison with fastANI (36). Therefore, we opted to investigate AAI in this case. The AAI values between strain FT118^T^ and other genomes were in the range of 44.22 to 58.53%, suggesting that it might represent a novel order (37). Also, according to GTDB-Tk, which normalizes rank with relative evolutionary divergence (38), strain FT118^T^ represents the first cultivated member of UBA8317, an uncultivated order proposed by GTDB. Within the UBA8317 cluster, strain FT118^T^ was placed on a separate branch, and it shared an AAI value of 54.51 to 56.33% with other UBA8317 genomes (Fig. S3). An uncultured alphaproteobacterium (GCA_009922025.1) shared an AAI value of 63.88 to 65.28% with the other eight UBA8317 members. The pairwise AAI result suggests that FT118^T^ and UBA8317 genomes possibly represent two divergent families within the proposed novel order, and one of the uncultured alphaproteobacteria (GCA_009922025.1) and the other eight members represent two genera within the same family (39).

Taken together, the above results strongly support that strain FT118^T^ and other UBA8317 genomes constitute an order-level lineage within *Alphaproteobacteria*, and within this order, strain FT118^T^ likely represents a family distinct from other UBA8317 genomes. Therefore, we propose *Futianiales* ord. nov. and *Futianiaceae* fam. nov. as a novel lineage within *Alphaproteobacteria*, and strain FT118^T^ represents the type strain of a novel species of a novel genus within *Futianiaceae*, for which the name *Futiania mangrovii* gen. nov., sp. nov. is proposed. Details of characterization are given in the taxonomic description section.

### Metabolic reconstruction of *Futiania mangrovii* FT118^T^.

Genome annotation revealed great potentials of diverse metabolism of strain FT118^T^. Complete pathways involved in essential cellular metabolism were identified. To verify whether these pathways were transcriptionally active in all sampled layers of mangrove sediments, the relative expression level of genes in these pathways were also evaluated.

**FIG 3:**
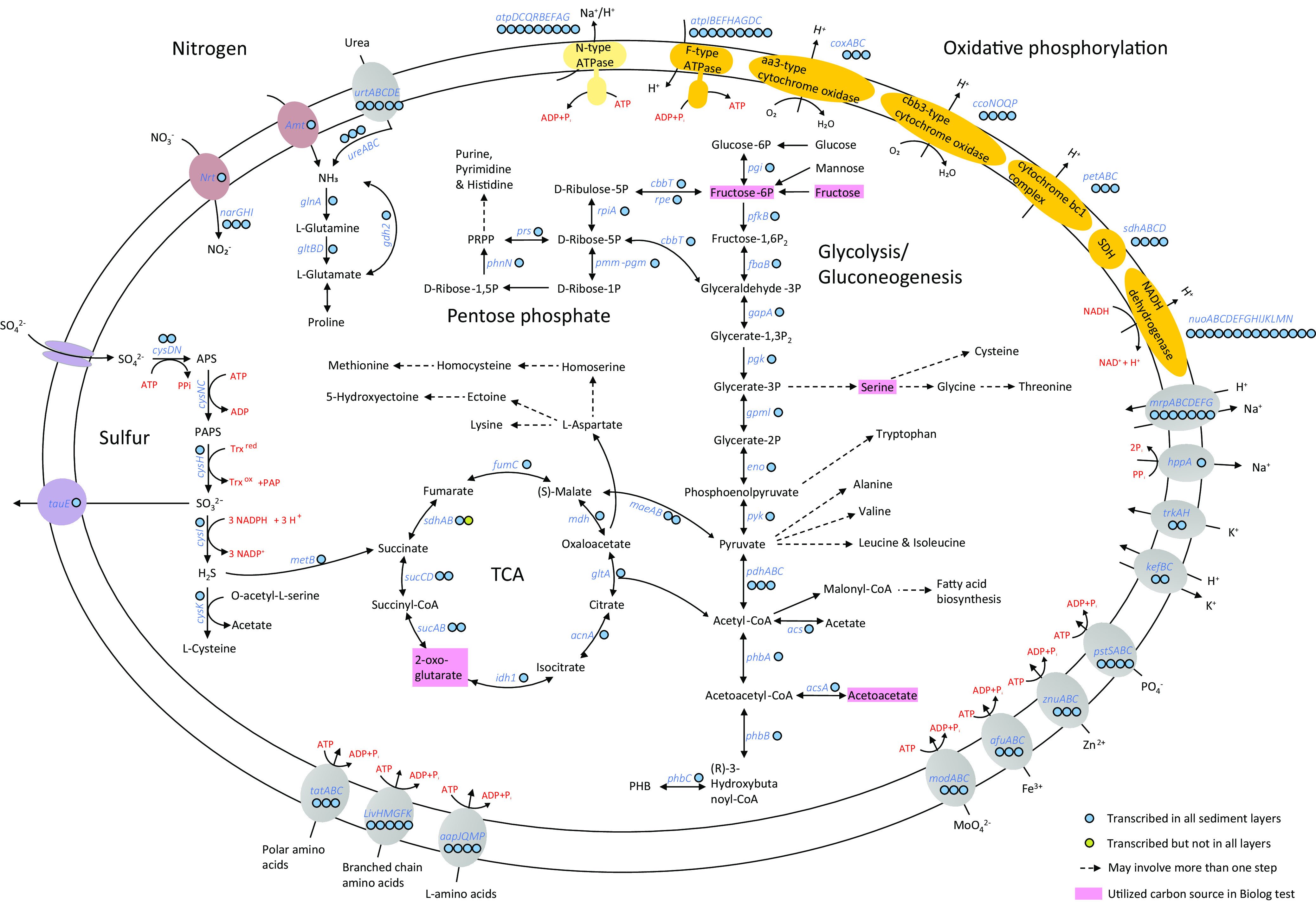
Metabolic reconstruction of *Futiania mangrovii* FT118^T^. Dashed arrows indicate that there may be more than one step in a pathway. Colored circles next to gene symbols indicate whether the genes were expressed in metatranscriptomic data. Detailed TPM values of genes involved in major metabolic pathways and adaptive features are listed in Table S4 in the supplemental material. Pink boxes highlight the carbon sources utilized by FT118^T^ in the Biolog GENIII test.

**(i) Carbon and energy metabolism.** Based on functional annotations of this novel lineage, strain FT118^T^ contains genes encoding key enzymes for essential carbohydrate metabolism (Fig. 3). It has a complete glycolysis (Embden-Meyerhof) pathway and a complete gluconeogenesis pathway, and its genome also encodes the pentose phosphate pathway and genes for phosphoribosyl diphosphate (PRPP) biosynthesis, which further enable purine, pyrimidine, and histidine metabolism. In addition, a complete tricarboxylic acid (TCA) cycle is present. In all the sampled layers of mangrove sediment, these pathways were both transcriptionally active, except that the transcription of the gene *sdhB* (iron-sulfur subunit of succinate dehydrogenase/fumarate reductase) was not detected in the 12 to 14 cm and 20 to 22 cm layers at MG1 site (Table S4). Although genes for glucose, mannose, and fructose utilization were found, only fructose was utilized. Glucose and mannose utilization were negative in the Biolog GENIII test. Genes for the utilization of other monosaccharides (allose, fucose, galactose, and rhamnose), disaccharides (maltose, sucrose, trehalose, and cellobiose) and polysaccharides (amylose, starch, and cellulose) were not detected in the genome. Of note, fucose and rhamnose utilization were weakly positive in the Biolog GENIII test (Table S1), despite that no complete pathway for these two substrates was confirmed in the genome, possibly due to unknown enzymes involved in the utilization process.

In terms of energy metabolism, the genome encodes a complete oxidative phosphorylation pathway (Fig. 3), including an NADH dehydrogenase (*nuoABCDEFGHIJKLMN*) for proton translocation, a succinate dehydrogenase (*sdhABCD*), a cytochrome *bc*_1_ complex (*petABC*), a heme *aa*_3_-type cytochrome *c* oxidase (*coxABC*), a *cbb*_3_-type cytochrome *c* oxidase (*ccoNOQP*), and an F-type ATPase (*atpIBEFHAGDC*). Besides the conventional F-type ATPase, we identified an N-type ATPase that might function as a Na^+^/H^+^ pump while consuming ATP. The above genes were expressed in all sediment layers except for *sdhB*. In addition, genes involved in polyhydroxybutyrate (PHB) synthesis (*phbABC*) and depolymerization (*phaZ*) were both identified and transcribed, among which the *phbB* gene was highly expressed (Table S4), indicating that PHB could be an important energy reserve for strain FT118^T^.

**(ii) Nitrogen, phosphorus, and sulfur metabolism.** Regarding nitrogen metabolism, genomic annotation predicted the presence of a nitrate/nitrite transporter (*NRT*) and a nitrate reductase (*narGHI*) that could convert nitrate to nitrite (Fig. 3). This process of nitrate reduction to nitrite was also demonstrated in the API 20NE test, although denitrification was not observed (Table S1). In addition, we found a copy of ammonium transporter (*amtB*) and ABC transporter for urea (*urtABCDE*). The urea could then be hydrolyzed by urease (*ureABC*). The resulting ammonia from these sources was used as a nitrogen source for amino acid synthesis, supported by the high expression level of glutamine synthetase (*glnA*) (Table S4). Like many members of *Alphaproteobacteria*, the enzyme activity of GlnA and GltBD is predicted to be regulated by the PII nitrogen response protein GlnB (40). Another PII protein GlnK-encoding gene was cotranscribed with the ammonium transporter (Table S4), which was commonly reported to regulate ammonium flux controlled by AmtB in a reversible way with changing ammonium levels (41, 42).

Besides the PII nitrogen sensor, strain FT118^T^ also encodes the Pho system in response to phosphate limitation. The Pho system comprises a high-affinity ABC transporter for phosphate (PstABCS) and a two-component signaling pathway (PhoR-PhoB). When the extracellular phosphate level becomes limiting, the phosphate uptake by the Pst transporter decreases, which stimulates PhoR to autophosphorylate and transfer the phosphoryl group to PhoB. After conformational changes, phosphorylated PhoB binds to conserved DNA sequences known as Pho boxes, resulting in increased transcriptional levels of target genes that cope with low extracellular phosphate conditions (43, 44). Furthermore, the API ZYM test result showed that FT118^T^ is positive in alkaline phosphatase activity and weakly positive in acid phosphatase activity (Table S1), suggesting that the strain is able to convert phosphorus-containing compounds to inorganic phosphate and release it into the environment (45).

The pathway of assimilatory sulfate reduction was identified in strain FT118^T^ (Fig. 3), where sulfate was initially transported by sulfate permease (*sulP*) and converted to adenosine 5′-phosphosulfate (APS) via *cysDN*, subsequently to 3′-phosphoadenosine 5′-phosphosulfate (PAPS) via *cysNC*, and then to sulfite via *cysH*. The produced sulfite could be either translocated by a sulfite exporter (*tauE*) or converted to sulfide via *cysI*. With the prediction of cysteine synthase (*cysK*), hydrogen sulfide could be utilized to produce l-cysteine. Alternatively, the reaction of hydrogen sulfide with *O*-succinyl-l-homoserine to produce l-homocysteine and succinate was allowed due to the presence of cystathionine gamma-synthase (*metB*). The above-mentioned genes were transcribed in different sediment layers (Fig. 3), suggesting that this strain is an active sulfur metabolizer *in situ*.

Strain FT118^T^ was predicted to be capable of synthesizing 18 amino acids, including alanine, aspartate, asparagine, glutamate, glutamine, serine, glycine, threonine, cysteine, methionine, valine, (iso)leucine, lysine, arginine, proline, histidine, and tryptophan (Fig. 3). Partial synthesis pathways were detected for tyrosine and phenylalanine, as the genome lacks chorismate mutase (EC 5.4.99.5) catalyzing the conversion between chorismate and prephenate. In addition to *de novo* synthesis, we recovered ABC transporters for general l-amino acids (*aapJQMP*), branched-chain amino acids (*livKHMGF*), and polar amino acids (ABC.PA.SPA). Additionally, we found many ABC transporters for metals such as molybdate (*modABC*), iron (*afuABC*), and zinc (*znuABC*) as well as for a variety of compounds, such as putrescine (*potFIHG*), signal peptide (*tatABC*), phosphate (*pstABCS*), lipoprotein (*lolCDE*), and lipopolysaccharide (*lptBFG*).

### Distribution of *Futianiales*.

**FIG 4:**
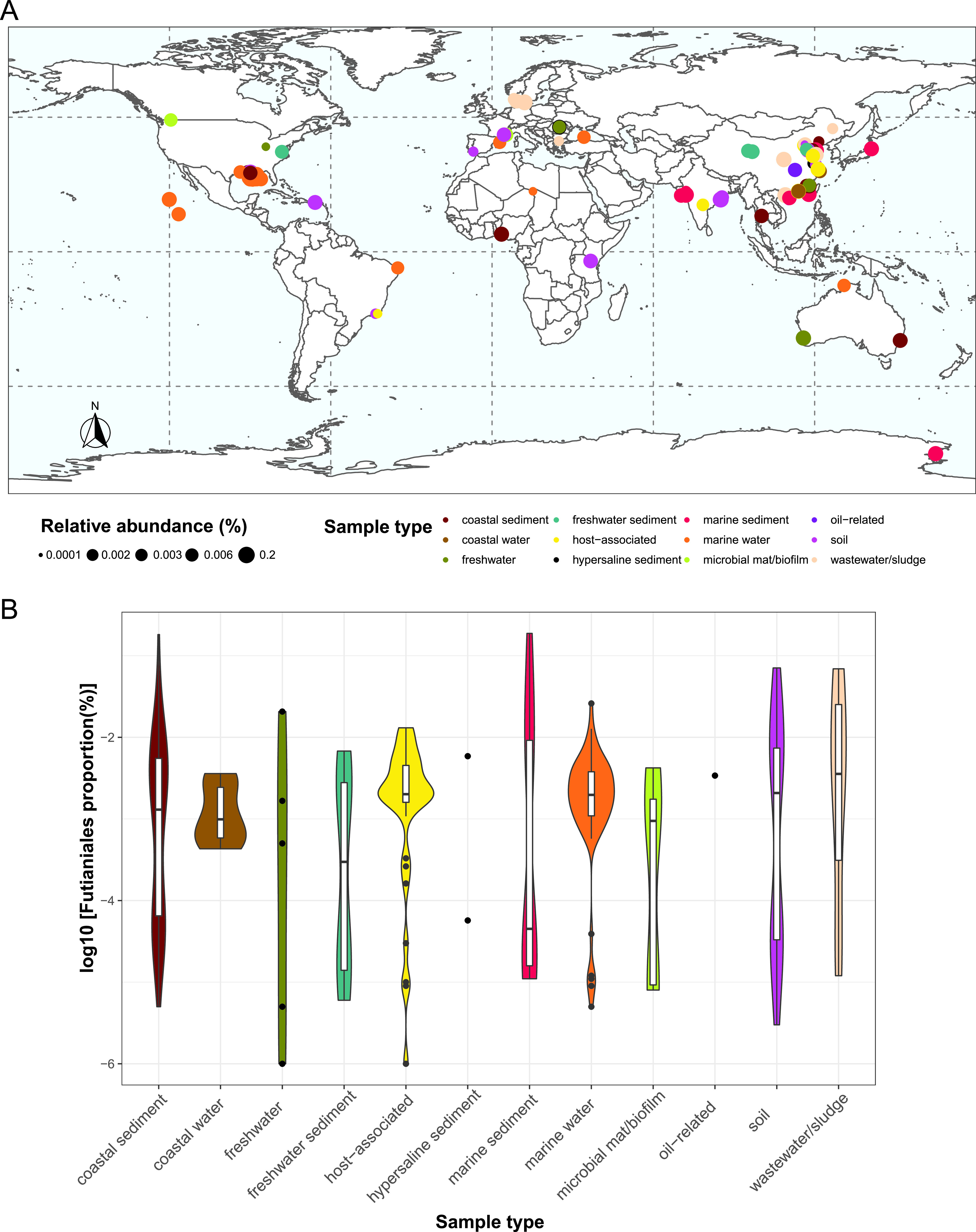
Global distribution of *Futianiales*. (A) Global distribution of *Futianiales* based on 268 16S rRNA gene amplicon data sets in which *Futianiales* members were present, as a query result from the IMNGS platform. (B) Relative abundance of *Futianiales* in different sample types. Detailed information of each amplicon data set can be found in Table S5 in the supplemental material. The world map was generated using R package rnaturalearth v.0.1.0 with R v.3.6.363.

The successful isolation of FT118^T^ and the proposed establishment of *Futianiales* prompted us to explore the global distribution of this novel order. We submitted four representative, nearly complete 16S rRNA gene sequences (the pink cluster containing the 16S rRNA gene sequence of FT118^T^ and the three most similar sequences identified from the Silva database displayed in Fig. 2A) to the integrated microbial next generation sequencing (IMNGS) platform with a sequence similarity cutoff of 97% (46). Overall, 268 16S rRNA gene amplicon data sets contained operational taxonomic units (OTUs) sharing at least 97% similarity with the submitted sequences of *Futianiales*. The results showed that members of this novel lineage were widely distributed across the globe and across various sample types, including the water body and sediment from coastal, freshwater, and marine ecosystems, hypersaline sediment, microbial mat/biofilm, soil, and wastewater treatment plants. Interestingly, it was also found in host-associated samples, such as human gastric mucosa, fish, and shrimp guts (Fig. 4A; Table S5). In general, *Futianiales* constituted a rare taxon in different environments, and its relative abundance varied significantly between different sample sites and even within the same sample type (Fig. 4B). Among 268 16S rRNA gene amplicon data sets from the IMNGS platform, *Futianiales* was the most abundant in two Bohai sea sediment samples (0.19% and 0.17%, respectively) and a coastal sediment in Louisiana (0.18%). This order had a relative abundance of >0.01% in just 32 amplicon data sets, and most frequently (236/268), its relative abundance was below 0.01% in the community (Table S5).

### Pangenome of *Futianiales*.

**FIG 5:**
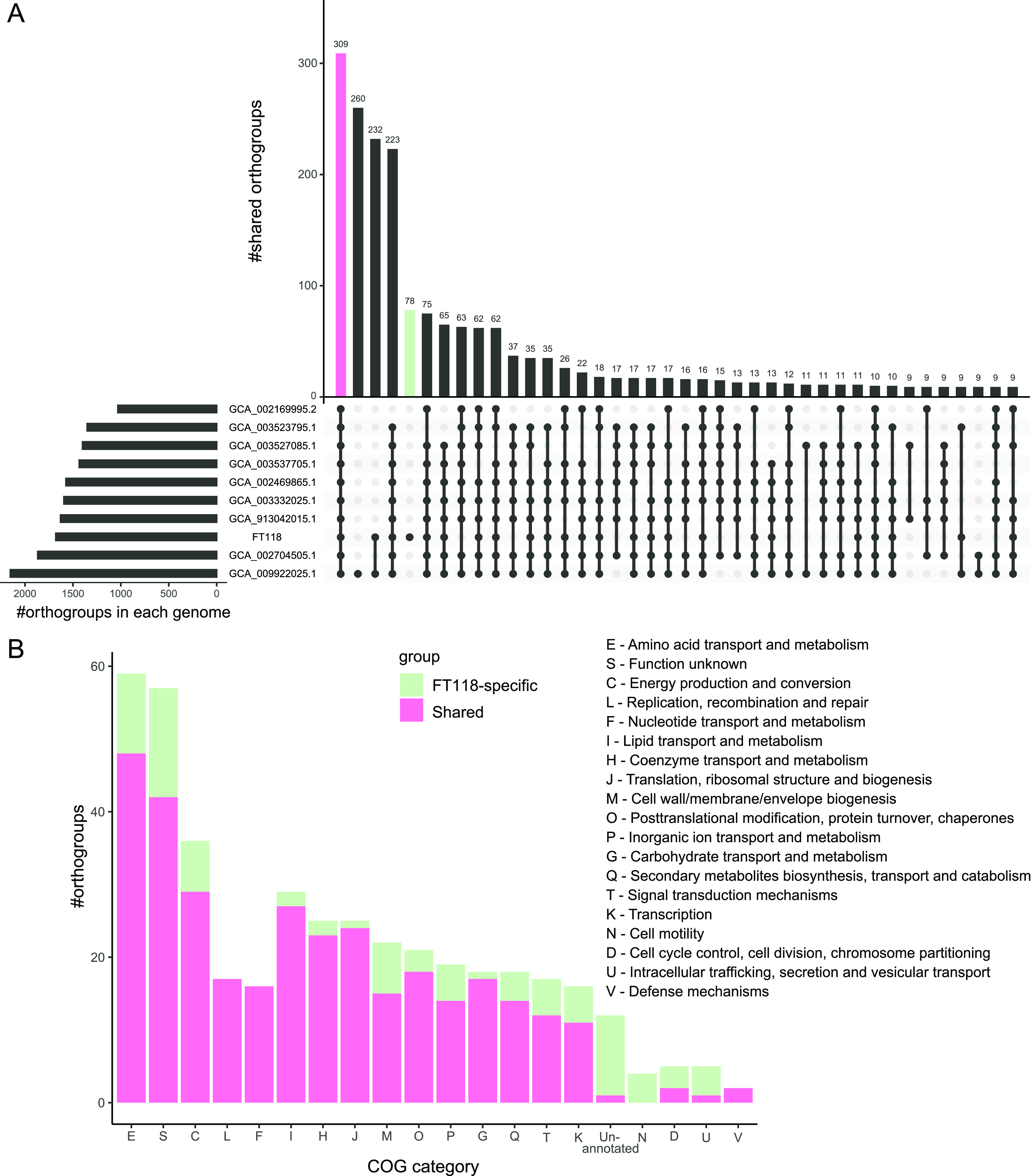
Pangenomic analysis of *Futianiales*. (A) Number of shared orthogroups between *Futianiales* genomes. (B) Functional classification of shared orthogroups and FT118-specific orthogroups according to the COG category.

The results of the pangenomic analysis obtained by OrthoFinder revealed a global pangenome of 2,516 orthogroups and a core genome of 309 orthogroups among 10 *Futianiales* genomes (Fig. 5A). These shared orthogroups were classified based on clusters of orthologous genes (COG) categories, and most were involved in central metabolism or housekeeping functions. The most frequently involved COG categories were amino acid transport and metabolism (E), function unknown (S), energy production and conversion (C), and lipid transport and metabolism (I) (Fig. 5B). However, 78 orthogroups appeared to be species specific in FT118^T^ and were not found in any other *Futianiales* genomes (Fig. 5A), most of which belonged to function unknown (S) or unannotated, amino acid transport and metabolism (E), energy production and conversion (C), and cell wall/membrane/envelop biogenesis (M) (Fig. 5B). These FT118-specific orthogroups included *cbb*_3_-type cytochrome *c* oxidase, N-type ATPase, and enzymes involved in assimilatory sulfate reduction (Table S6), which likely contribute to the environmental adaptability of this novel alphaproteobacterium.

### Genomic adaptations of *Futianiales* to fluctuating environments.

Mangrove ecosystems experience considerable daily and seasonal changes in temperature, pH, and salinity due to the rainy/dry season, high/low tide, and sometimes freshwater runoff from inland (47, 48). To survive in this rapidly changing environment, mangrove organisms have developed energy-consuming mechanisms to counterbalance water and ion fluxes. In laboratory settings, strain FT118^T^ is tolerant to a wide range of temperatures (20 to 45°C), pH values (6 to 10), and salinity conditions (0 to 13%). Moreover, a sequence similarity search against available 16S rRNA gene amplicon data sets revealed a ubiquitous distribution of *Futianiales* worldwide. This evidence suggests that members of this novel order can physiologically adapt to various environments, which brings us to look further into its putative adaptive features.

**(i) Cytochrome *c* oxidases.** The oxidative phosphorylation pathways of strain FT118^T^ and other *Futianiales* genomes are almost identical, except that strain FT118^T^ encodes one *aa*_3_-type cytochrome *c* oxidase and one *cbb*_3_-type cytochrome *c* oxidase as part of the electron transport chain, while the latter is not found in any other *Futianiales* genomes (Table 2). Generally, the *cbb*_3_-type cytochrome *c* oxidase has a high affinity for O_2_, and the *aa*_3_-type oxidases are low-affinity oxidases (49). Both types are widely distributed in diverse taxa and are also the most common terminal oxidases among *Alphaproteobacteria*, in which many members have different combinations of terminal oxidases. For instance, containing both high- and low-affinity oxidases is a common trait in many *Rhodobacterales* and *Rhodospirillales* members. In comparison, some other *Rhodospirillales* members have high-affinity oxidases only, whereas some members of *Rickettsiales* and *Pelagibacterales* have low-affinity oxidases only (49). In the facultative *Rhodobacterales* bacterium Rhodobacter sphaeroides, it has been demonstrated that the *aa*_3_-type cytochrome *c* oxidase is more active under aerobic conditions, while the expression of the *cbb*_3_-type cytochrome *c* oxidase occurs mainly under microaerobic conditions (50). A similar phenomenon was also observed in Azospirillum brasilense that the *cbb*_3_-type cytochrome *c* oxidase was required for growth under microaerobic conditions (51). This leads to an inference that other *Futianiales* members are possibly obligate aerobes, with the *aa*_3_-type cytochrome *c* oxidase being the sole terminal oxidase. In contrast, encoding terminal oxidases of various affinities in strain FT118^T^ supports the observation of limited growth under anaerobic conditions.

**TABLE 2:** Comparison of genomic content of FT118^T^ and other *Futianiales* genomes

Pathway/Feature	*Futiania mangrovii* FT118^T^^*a*^	Other *Futianiales* genomes^*a*^
Glycolysis	+	p
TCA	+	p
Pentose phosphate	+	+
Oxidative phosphorylation		
NADH dehydrogenase (*nuoABCDEFGHIJKLMN*)	+	+
Succinate dehydrogenase (*sdhABCD*)	+	+
Cytochrome *c* reductase (*petABC*)	+	p
Cytochrome *c* oxidase-*aa*_3_ type (*coxABC*)	+	+
Cytochrome *c* oxidase-*cbb*_3_ type (*ccoNOQP*)	+	−
F-type ATPase (*atpIBEFHAGDC*)	+	+
N-type ATPase (*atpDCQRBEFAG*)	+	−
PHB synthesis	+	+
Nitrate reduction to nitrite	+	−
Assimilatory sulfate reduction	+	−
Sulfur oxidation	−	+
Osmolyte system		
Alanine synthesis (EC 2.6.1.21, EC 5.1.1.1, EC 1.4.1.1)	+	p
Ectoine/hydroxyectoine synthesis (*ectABC*)	+	−
Ectoine/hydroxyectoine transport (*ehuABCD*)	p	−
Glutamate synthesis (*gltBD*)	+	+
Glutamate transport (*gltIKJL*, *gluABCD*)	−	−
Glutamine synthesis (EC 6.3.1.2)	+	+
Glutamine transport (*glnHPQ*, *bgtAB*)	−	−
Glycine betaine synthesis (*betAB*)	p	p
Glycine betaine/proline transport (*proVWX*)	−	+
Proline synthesis (*proABC*)	+	p
*N*-∂-Acetyl-ornithine synthesis (*argJBCD*)	+	p
Betaine/carnitine/choline transporter (BCCT)	+	−
Sodium pump (*hppA*)	+	−
Na^+^:H^+^ antiporter (*nhaA*)	−	+
Multicomponent Na^+^:H^+^ antiporter (*mrpABCDEFG*)	+	−
Glutathione-regulated potassium-efflux system (*kefBC*)	+	−
K^+^ uptake channel (*trkAH*)	+	+

a For these *Futianiales* genomes, + represents more than half of the genomes encode the pathway/enzyme, − represents more than half of the genomes do not encode the pathway/enzyme, and p represents more than half of the genomes encode the partial pathway/enzyme.

**(ii) N-type ATPase.** In addition to a copy of F-type ATPase responsible for H^+^ translocation and ATP synthesis (52), the genome of strain FT118^T^ encodes one copy of N-type ATPase, which is another FT118-specific feature in *Futianiales* (Table 2). There are nine open reading frames in this N-type *atp* operon. The operon organization is the same as those detected in closely related and distant bacterial and archaeal taxa (*atpDCQRBEFAG*), including members of *Pseudomonadota*, *Aquificota*, *Chlorobiota*, *Plantomycetota*, and a few archaea (53). Compared to the conventional F-type *atp* operon, the β-subunit (*atpD*) and ε-subunit (*atpC*) are shifted to the N terminus of the N-type *atp* operon. Following the first two subunits are *atpQ* and *atpR*, which are not present in the conventional F-type *atp* operon. The *atpR* gene is considered a distinctive feature of the N-type ATPase. The function of this gene product may be regulating the assembly and/or activity of the N-type ATPase via the interaction of two arginine residues with the *c*-subunits specific to the N-type ATPase, where two glutamate residues are commonly found in the middle of the transmembrane helices (53). When searching in the complete genome database, it was found that the N-type ATPases always cooccur as additional copies alongside the conventional ones and represent a separate branch from the F-type ATPases (53). Furthermore, structural and biochemical studies show that N-type ATPases could translocate Na^+^ (54) or H^+^ (55), suggesting that the N-type ATPases represent an early diverging branch likely resulting from horizontal gene transfer and function as ATP-driven ion pumps, which may contribute to salt or acid stress tolerance. Encoded by *atpE*, the *c*-subunit of the N-type ATPase in strain FT118^T^ contains a glutamic acid residue in the N-terminal helix region, which putatively functions as a Na^+^ ligand; but, it lacks the typical “ESTxxY” motif for Na^+^ binding in the C-terminal helix (Fig. S4), similar to that reported in the betaproteobacterial pathogen Burkholderia pseudomallei (55), suggesting that this copy of N-type ATPase may be predominantly H^+^ selective and use the H^+^ gradient across bacterial membrane.

**(iii) Osmolyte system.** In mangrove sediments where a salinity gradient is observed with increasing depths, microorganisms are required to grow and deal with fluctuations in salinity to maintain an osmotic balance. A small number of halophiles use the “salt-in” strategy, which involves the accumulation of Cl^−^ and K^+^ in the cell, while a more common “salt-out” strategy adopted by microorganisms is to extrude Na^+^ from the cytoplasm and accumulate high concentrations of compatible solutes (56). These are small organic molecules, including polyols, sugars, amino acids, and their derivatives, that protect cellular components under unfavorable conditions via biosynthesis and/or transport into the cell (57). As FT118^T^ is halotolerant and grows in high salinities, we calculated the isoelectric points of the predicted proteome of each *Futianiales* genome. The average isoelectric points of *Futianiales* members range from 6.5 to 6.8, and their profiles are similar to the salt-out halophiles (Fig. S5), indicating that they accumulate compatible solutes to counterbalance osmotic stress (58). Next, we investigated the osmolyte system of *Futianiales* members based on the genomic annotation result (Table 2). Specifically, the genome of strain FT118^T^ encodes key genes involved in the biosynthesis of alanine, ectoine/hydroxyectoine, glutamate, glutamine, *N*-∂-acetyl-ornithine, and proline. These amino acids are widely present in *Alphaproteobacteria* (59), and their production/import has been demonstrated to support bacterial growth under osmotically challenging conditions (60). Although FT118^T^ cannot synthesize another three common amino acid derivatives (taurine, choline, and glycine betaine), it encodes a copy of the betaine/carnitine/choline transporter, a copy of the general l-amino acid transporter, and multiple copies of branched-chain amino acid transporters and putative polar amino acid transporters. As for other members of *Futianiales*, a major difference in the compatible solute pool is the lack of genomic capability of synthesizing ectoine/hydroxyectoine and the presence of the glycine betaine/proline transporter (*proVWX*) (Table 2).

Besides these low-molecular-weight organic molecules acting as osmoprotectants, cation transmembrane transporters play a pivotal role in regulating pH and ion homeostasis under saline and alkaline conditions (61). The strain FT118^T^ genome encodes a copy of a K^+^-stimulated pyrophosphate-energized sodium pump (*hppA*) and a copy of the multicomponent Na^+^:H^+^ antiporter (*mrpABCDEFG*). Belonging to the large subfamily of K^+^-dependent pyrophosphatases (PPases), the sodium pump HppA is a primary pump that translocates Na^+^ or H^+^ in some bacteria and archaea (62). In mesophilic Moorella thermoacetica, this sodium pump potentially works in conjunction with other Na^+^:H^+^ antiporters to maintain the cellular Na^+^ levels when growing at high salinities (63). In comparison, the thermophilic Thermotoga maritima uses the Na^+^-PPase together with ATP synthase for energy to maintain the sodium gradient, particularly when energy is limiting (64). Widely distributed in bacteria and archaea, the Mrp antiporter is recently classified as the monovalent cation:proton anitoporter-3 (CPA3) family (65). In alkaliphilic *Bacillus* spp. and *Halomonas* spp., the Mrp complex exports Na^+^ and simultaneously imports H^+^, enhancing sodium tolerance and pH homeostasis under highly alkalisaline conditions (66). Compared with strain FT118^T^, the other *Futianiales* genomes appear to contain the Na^+^:H^+^ antiporter *nhaA*, which promotes bacterial adaptation to high salinity and alkaline environments (67, 68), as substitutions to the sodium pump HppA and the Mrp complex in strain FT118^T^.

Cytoplasmic K^+^ accumulation represents an important strategy in many alkaliphilic microorganisms to respond to Na^+^ toxicity (69). We identified a copy of the glutathione-regulated potassium efflux system (*kefBC*) and a copy of the K^+^ uptake channel (*trkAH*) in the strain FT118^T^ genome. In comparison, other *Futianiales* genomes lack the Kef K^+^ efflux system (Table 2). The Kef system is a K^+^: H^+^ antiporter present in most Gram-negative pathogens (70). When electrophiles react with glutathione, the formed glutathione-*S*-conjugates activate the Kef system, which causes K^+^ efflux accompanied by H^+^ influx and thus results in decreased cellular pH and detoxification of toxic glutathione-*S*-conjugates to less toxic species, protecting bacterial cells from damaging electrophiles (71). The TrkAH complex is formed by the assembly of the K^+^ transporter TrkH with its regulatory protein TrkA (72), catalyzing the uptake of K^+^ and possibly concomitant H^+^ import, therefore contributing to cellular K^+^ and pH homeostasis in halophilic and/or alkaliphilic microorganisms (73). Altogether, these results suggest that members of *Futianiales* adopt the salt-out strategy with slight variations in osmolyte systems.

**(iv) PHB synthesis.** PHB is a type of polyhydroxyalkanoate (PHA) belonging to the class of polyesters. It is a biodegradable plastic naturally synthesized by microorganisms. PHB synthesis starts from condensating two acetyl-coenzyme A (acetyl-CoA) molecules into acetoacetyl-CoA catalyzed by β-ketothiolase (*phbA*). Then, acetoacetyl-CoA reductase (*phbB*) catalyzes the reduction process from acetoacetyl-CoA to *R*-3-hydroxybutyryl-CoA, followed by the polymerization of *R*-3-hydroxybutyryl-CoA to PHB with the action of PHB synthase (*phbC*) (74). Under energy-limiting conditions, PHB can be hydrolyzed as a carbon and energy source, which is catalyzed by PHB depolymerase (*phaZ*). In the genome of strain FT118^T^, these genes were both detected and transcribed in both surface and deep sediment layers (Table S4), indicating that this pathway was active, and PHB could be a nutrient source for strain FT118^T^ when necessary. Additionally, more than half of other *Futianiales* genomes can potentially produce and degrade PHB (Table 2). PHB production has been demonstrated in diverse taxa, such as alphaproteobacterial members *Rhodobacter*, *Rhodospirillum*, *Rhizobium*, and *Pseudodonghicola*, betaproteobacterial members *Ralstonia* and *Alcaligenes*, and gammaproteobacterial members Pseudomonas (75). In different taxa, PHB production can be strongly affected by carbon and nitrogen sources, C/N ratio, temperature, and incubation time (76, 77). Starvation experiments showed that *Sinorhizobium melilot* populations with high cellular PHB content not only reproduced significantly more in the initial 29 to 36 days when most of the stored PHB was consumed but also survived much longer than populations with low PHB during a starvation period of 160 days (78). Therefore, having this trait can potentially increase short-term fitness of *Futianiales* members by providing essential carbon and energy sources when environmental nutrients are scarce, for instance in deep sediments.

The metatranscriptomic data show that genes involved in major pathways and those encoding the above-mentioned adaptive features were both expressed in all sampled sediment layers (Table S4). We also performed Spearman correlation analysis between the expression level of major metabolic pathways/adaptive features and measured environmental variables (Fig. S6). In general, the expression of oxidative phosphorylation was the highest among all major pathways, and it showed a significant positive correlation with depth (*P* < 0.01), pH (*P* < 0.01), salinity (*P* < 0.05), and NO_3_^−^-N (*P* < 0.05), suggesting a pivotal role of energy production via this pathway in deeper sediments. Additionally, depth, pH, and salinity were significantly positively correlated with the expression of the pentose phosphate pathway and nitrogen-related metabolism. Moreover, the expression of pyruvate metabolism and the TCA cycle negatively correlated with NH_4_^+^-N (*P* < 0.01). In terms of the osmolyte system, we did not observe a correlation between the transcripts per million (TPM) values of the above-mentioned adaptive features and salinity. However, as salinity gets higher with increasing sediment depth, the expression of amino acid transporters, ectoine/hydroxyextoine and proline synthesis, K^+^ uptake channel (*trkAH*), and sodium pumps (Mrp complex and *hppA*) reached the highest in the deepest sediment (28 to 30 cm) (Table S4). Altogether, our metatranscriptomic data show that the major metabolic pathways of FT118^T^ were active in different sediment layers, and the expression of its adaptive features changed under various environmental conditions. Further transcriptome and metabolome analyses would be required to elucidate its adaptation to environmental fluctuations *in situ*.

### Conclusions.

This study presents the first cultured member of the novel order *Futianiales* of *Alphaproteobacteria*. We show that this order has a ubiquitous distribution across various habitats but is a rare member in all different types of environments worldwide. Sequence analysis and experimental validation conclude that this taxon can physiologically adapt to a wide range of oxygen levels, temperatures, pH values and salinity levels. Biochemical characterization and genomic and metatranscriptomic analyses revealed that strain FT118^T^ exhibits diverse metabolic potentials, and its central metabolic pathways are transcriptionally active in sampled mangrove sediments. Some of its adaptive features are distinct from other *Futianiales* genomes, possibly allowing for the persistence and growth of this novel taxon across various habitats. Collectively, these results expand our current culture collections of mangrove microorganisms, elucidate how this new alphaproteobacterium adapts to fluctuating mangrove environments, and provide a reference for future ecological and taxonomical studies.

Based on the combination of phylogenetic, physiological, and biochemical traits of strain FT118^T^, we propose the first family *Futianiaceae* fam. nov. within a novel order *Futianiales* ord. nov. (previously uncultured order UBA8317) and strain FT118^T^ to be the type strain of a novel species of a novel genus *Futiania* gen. nov. from this family for which *Futiania mangrovii* sp. nov. was named.

### Description of *Futiania* gen. nov.

*Futiania* (Fu.ti.an’ia. L.fem. n. *Futiania* referring to the origin of this organism, Futian National Natural Reserve).

Cells are Gram-stain-negative, facultative aerobic, motile, and ovoid to rod shaped and catalase and oxidase positive. The predominant isoprenoid quinone is ubiquinone Q10. Major cellular fatty acids are C18:1 ω7*c*, C19:0 cyclo ω8*c*, and C16:0. Polar lipids include diphosphatidylglycerol (DPG), phosphatidylethanolamine (PE), phosphatidylmethylethanolamine (PME), an unidentified aminolipid (AL), an unidentified aminophospholipid (APL), and an unidentified phospholipid (PL). The type species is *Futiania mangrovii*, which was isolated from mangrove sediment of Futian National Natural Reserve.

### Description of *Futiania mangrovii* sp. nov.

*Futiania mangrovii* (man.gro.vi’i. L. n. from mangrove).

Colonies are white, circular, smooth, and raised after growing on 2216 agar at 37°C for 4 days. Cells are Gram-stain-negative, facultative aerobic, motile, and ovoid to rod shaped. Growth occurs at 20 to 45°C (optimum of 30 to 40°C) and a pH of 6 to 10 (optimum of 6 to 7) and with 0 to 13% NaCl (optimum of 2%). Nitrate is reduced to nitrite but not to nitrogen. Indole is not produced. Glucose is not fermented. Production of arginine dehydrolase is negative. Gelatin is hydrolyzed but not esculin. Production of alkaline phosphatase, esterase (C4), esterase lipase (C8), leucine acrylamidase, valine acrylamidase, and naphthol-AS-BI-phosphohydrolase is positive. Production of acid phosphatase is weakly positive. Production of lipase (C14), cystine acrylamidase, trypsin, α-chymotrypsin, α-galactosidase, β-galactosidase, β-glucuronidase, α-glucosidase, β-glucosidase, *N*-acetyl-β-glucosaminidase, α-mannosidase, and α-fucosidase is negative. Utilization of *N*-acetyl neuraminic acid, myo-inositol, d-fructose-6-P, and glucuronamide is positive. Utilization of dextrin, d-turanose, d-fructose, d,l-fucose, l-rhamnose, d-serine, l-histidine, d-galacturonic acid, l-galactonic acid lactone, d-glucuronic acid, d-saccharic acid, α-keto-glutaric acid, l-malic acid, and acetoacetic acid is weakly positive. Utilization of other substrates in the Biolog GENIII test is negative. The DNA G+C content of the type strain is 68.5% (by genome).

The type strain is FT118^T^ (=MCCC 1K07814^T^=KCTC 92476^T^), isolated from a mangrove sediment sample from Futian National Natural Reserve in Shenzhen, China, and deposited at Marine Culture Collection of China and Korean Collection for Type Cultures.

### Description of *Futianiaceae* fam. nov.

*Futianiaceae* (Fu.ti.an.ia.ce’ae. L. fem. n. *Futiania* type genus of the family; suff. -aceae ending to denote a family; L. fem. pl. n. *Futianiaceae*, the *Futiania* family).

At present, the family *Futianiaceae* is composed of solely the genus *Futiania*, which is a novel family of the order *Futianiales* (previously uncultured UBA8317). The description of this novel family is the same as that given above for the type genus *Futiania*.

### Description of *Futianiales* ord. nov.

*Futianiales* (Fu.ti.an.ia’les. L. fem. n. *Futiania* type genus of the order; -ales ending to denote an order; L. fem. pl. n. *Futianiales* the order of the genus *Futiania*).

The description of this novel order is the same as that given above for the type genus *Futiania*.

## MATERIALS AND METHODS

### Sample collection, chemical analysis, and isolation.

Mangrove sediment samples were taken at Futian Mangrove Natural Reserve (22°31′35″N, 114°1′34″E) in Shenzhen, China, in July 2020. All sediment samples were kept in sterile plastic bags in a precooled container and transported to the laboratory immediately. Measurement of physiochemical parameters of sediment was performed according to a previous study (79). About 2 g of sediment sample was taken from the 0- to 10-cm layer and serial diluted with sterile saline. Each dilution was spread onto 2216 agar (Hopebio) and incubated at 37°C in the dark for 2 weeks. Colonies were streaked repeatedly until pure cultures were obtained. Strain FT118^T^ was maintained on 2216 plates at 4°C and preserved with 25% (vol/vol) glycerol at −80°C. Strain FT118^T^ was also deposited at Marine Culture Collection of China (MCCC) and Korean Collection for Type Cultures (KCTC) under the accession numbers MCCC 1K07814 and KCTC 92476, respectively.

### Nucleic acid extraction, whole-genome sequencing, assembly, and annotation.

Genomic DNA was extracted from 2216 liquid culture of strain FT118^T^ using a TaKaRa MiniBEST bacteria genomic DNA extraction kit version 3.0 (TaKaRa). DNA quality was examined by gel electrophoresis and optical density OD_260_/OD_280_ ratio measured by a NanoDrop 2000 spectrophotometer (Thermo Fisher Scientific). Whole-genome shotgun sequencing was performed at Novogene Bioinformatics Technology Co., Ltd. (Tianjing, China), on an Illumina HiSeq using the PE150 strategy (paired-end reads of 2 × 150 bp). Quality filtering and trimming of the raw reads was performed with Trimmomatic (80), followed by *de novo* assembly using SPAdes v3.15.1 (81). Quality assessment of the assembly was done by CheckM v1.1.3 (82). Gene prediction was performed using Prodigal v2.6.3 (83). Functional annotation was done with different tools by comparing with various databases, including eggNOG-mapper v2.0 (search against eggNOG v5.0 database) (84), hmmsearch against the COG database (the 2020 release) (85), KEGG BlastKOALA (86), and InterProScan Version 5.52 to 86.0 (87). The isoelectric point of the predicted proteins was calculated using an online protein isoelectric point calculator (http://www.endmemo.com/bio/proie.php).

### Phylogenetic, phylogenomic, and pangenomic analyses.

For the whole-genome-based phylogeny, genome sequences of all type species within *Alphaproteobacteria* were downloaded from NCBI RefSeq database (*n* = 1,621, as of June 2021). A phylogenomic tree was inferred by IQTree 2 (88) based on the concatenated alignment of 120 conserved proteins produced by GTDB-Tk (38) under the best-fit model LG+F+R10 and assessed with 1,000 samples for ultrafast bootstrap (UFBOOT) and 1,000 replicates for SH-like approximate likelihood ratio test (SH-aLRT). For the 16S rRNA gene phylogeny, PCR was performed to target the full-length 16S rRNA gene of strain FT118^T^ using a universal primer pair 27F (5′ to 3′ AGAGTTTGATCMTGGCTCAG) and 1492R (5′ to 3′ TCAGGYTACCTTGTTACGACTT). The 16S rRNA gene sequences of other alphaproteobacterial type strains were extracted from their genomes using Barrnap (https://github.com/tseemann/barrnap). If more than two 16S rRNA gene sequences were present in a genome, the distance between each copy was calculated under the “identity” model in biopython, and the copy with the shortest distance to all other copies was chosen for analysis; otherwise, 16S rRNA gene sequences were selected randomly (89) and aligned using MAFFT version 7 (90). The alignment was trimmed with ClipKIT v1.1.5 (91). A maximum likelihood tree was then inferred by IQTree 2 (88) under the best-fit model GTR+F+R10 and assessed with 1,000 UFBOOT and 1,000 SH-aLRT replicates. The trees were visualized and annotated using Figtree v1.4.4 (http://tree.bio.ed.ac.uk/software/figtree/).

The pairwise average nucleotide identity (ANI) and average amino acid identity (AAI) were calculated between the genomes of *Futianiales* and genomes of type species within *Alphaproteobacteria* using FastANI (36) and the AAI workflow of CompareM v0.1.2 (https://github.com/dparks1134/CompareM), respectively. First, the genome of strain FT118^T^ was compared against genomes of type species from each alphaproteobacterial orders separately. Then, five genomes of each order that ranked at 0, 25, 50, 75, and 100% in terms of AAI value were selected and computed again pairwisely with the genomes of *Futianiales* to generate the figure.

Pangenomic analysis was performed between strain FT118^T^ and other *Futianiales* genomes using OrthoFinder v2.5.4 (92). The functional classification of their orthogroup sequences were based on the COG database (the 2020 release) (85).

### Metatranscriptomic sequencing and gene expression.

Samples from five layers (0 to 2 cm, 6 to 8 cm, 12 to 14 cm, 20 to 22 cm, and 28 to 30 cm) were selected for metatranscriptomic sequencing. Total RNA was isolated from 10 to 20 g of sediment using an RNeasy PowerSoil total RNA kit (Qiagen). Genomic DNA was removed using a Turbo DNA-free kit (Ambion), and rRNA was removed using a Ribo-Zero removal kit (Illumina). The purified RNA was sequenced at Novogene Bioinformatics Technology Co., Ltd. (Tianjing, China), on an Illumina HiSeq 2000 using the PE150 strategy (paired-end reads of 2 × 150 bp). Approximately 220 gigabase pairs (Gbp) of raw sequence data were obtained for each sample.

Raw reads from metatranscriptomic sequencing were quality trimmed and filtered using Sickle (https://github.com/najoshi/sickle). SortMeRNA v4.3.4 (93) was used to filter rRNA and tRNA fragments from metatranscriptomic data. The paired-end reads from metatranscriptomic sequencing were mapped onto the predicted gene sequences of strain FT118^T^ using BWA mem (94) with the default setting. The coverage information was extracted using SAMtools v1.3.1 (95) and bedtools v2.30.0 (96). The transcript per million (TPM) values were calculated to determine gene expression activity in metatranscriptomes.

### Morphological, physiological, biochemical, and chemotaxonomic characterization.

The cell morphology of strain FT118^T^ was observed with a transmission electron microscope (HT7700 Exalens, Hitachi, Japan) using cells freshly cultured in 2216 broth (Hopebio) and negatively stained using phosphotungstic acid.

All biochemical and physiological tests were conducted with strain FT118^T^ grown on 2216 medium to the log phase. Gram staining was performed using a Gram staining kit (Hopebio). The oxygen requirement of the strain was tested by observing its growth in an anaerobic chamber (Bactron EZ-2, Shellab, USA) within 2 weeks. Mobility was tested using semisolid 2216 medium. The temperature range and the optimal temperature for bacterial growth was measured with cultures incubated at 5 to 55°C in 2216 broth. The pH range for growth was tested within a pH range of 5 to 10 at 37°C in 2216 broth. NaCl tolerance was tested by growing in 2216 broth with 0 to 14% NaCl (wt/vol, in increments of 1%).

Catalase activity was tested by observing bubble production in 3% hydrogen peroxide solution. Oxidase activity was examined using an oxidase test strip (Hopebio). Hydrolysis of casein, starch, and cellulose was tested using skim milk agar, starch agar, and carboxymethylcellulose agar, respectively. Enzyme activities were examined with the API ZYM test (bioMérieux). Acid production from various carbohydrates was tested with the API 50CH test (bioMérieux). API 20NE (bioMérieux) was also performed to test other biochemical properties. Carbon utilization was examined using the Biolog GENIII microplate system (Biolog). All these tests were performed according to the manufacturers’ instructions.

Cellular fatty acids were extracted using the MIDI protocol (Sherlock Microbial Identification System version 6.3) and analyzed by gas chromatography (Agilent Technologies, 6850). The polar lipid content of strain FT118 was analyzed by two-dimensional thin-layer chromatography (TLC). Different staining solutions were used to spray the plate, including ninhydrin for detection of aminolipids, molybdenum blue for detection of phospholipids, ethanolic phosphomolybdic acid for detection of total lipids, Dragendorff’s reagent, and α-naphthol. Chloroform/methanol/water (65:25:4 [vol/vol/vol]) was applied in the first direction, and chloroform/acetic acid/methanol/water (80:18:12:5 [vol/vol/vol/vol]) was applied in the second direction. Quinone was extracted and separated into different classes by TLC and further analyzed by high-performance liquid chromatography (97).

### Data availability.

The GenBank/EMBL/DDBJ accession numbers for the 16S rRNA gene sequence and the draft genome sequence of strain FT118^T^ are ON876767 and JAMZFT000000000, respectively. In addition, the 16S rRNA sequence and genome sequence from the current study have been deposited in an eLibrary of Microbial Systematics and Genomics (eLMSG; https://www.biosino.org/elmsg/index) under accession numbers LMSG_R000000133.1 and LMSG_G000011400.1 (https://www.biosino.org/elmsg/record/MSG083698), respectively. The metatranscriptomic data have been deposited in National Omics Data Encyclopedia (NODE; https://www.biosino.org/node/) under project ID OEP001892 (experiment ID OEX012602).
