# Supplementary material for: Isolation and Genomics of Futiania mangrovii gen. nov., sp. nov., a Rare and Metabolically Versatile Member in the Class Alphaproteobacteria
Source: Microbiol Spectr. 2022 Dec 21;11(1):e04110-22. doi: 10.1128/spectrum.04110-22 (PMC9927469; doi:10.1128/spectrum.04110-22)
Supplement: Supplemental file 1 — Fig. S1-S6 and Tables S1-S7. Download spectrum.04110-22-s0001.pdf, PDF file, 2.5 MB [file spectrum.04110-22-s0001.pdf]

Supplemental Material for Publication

Isolation and Genomics of *Futiania mangrovii* gen. nov., sp. nov., a Rare and Metabolically-versatile Member in the Class Alphaproteobacteria

Lirui Liu, Wen-Cong Huang, Jie Pan, Jiayi Li, Yuhan Huang, Huan Du, Yang Liu, Meng Li*

*Corresponding author:

Prof. Meng Li, limeng848@szu.edu.cn

This PDF file includes Figure S1-S6 and Table S1-S7.

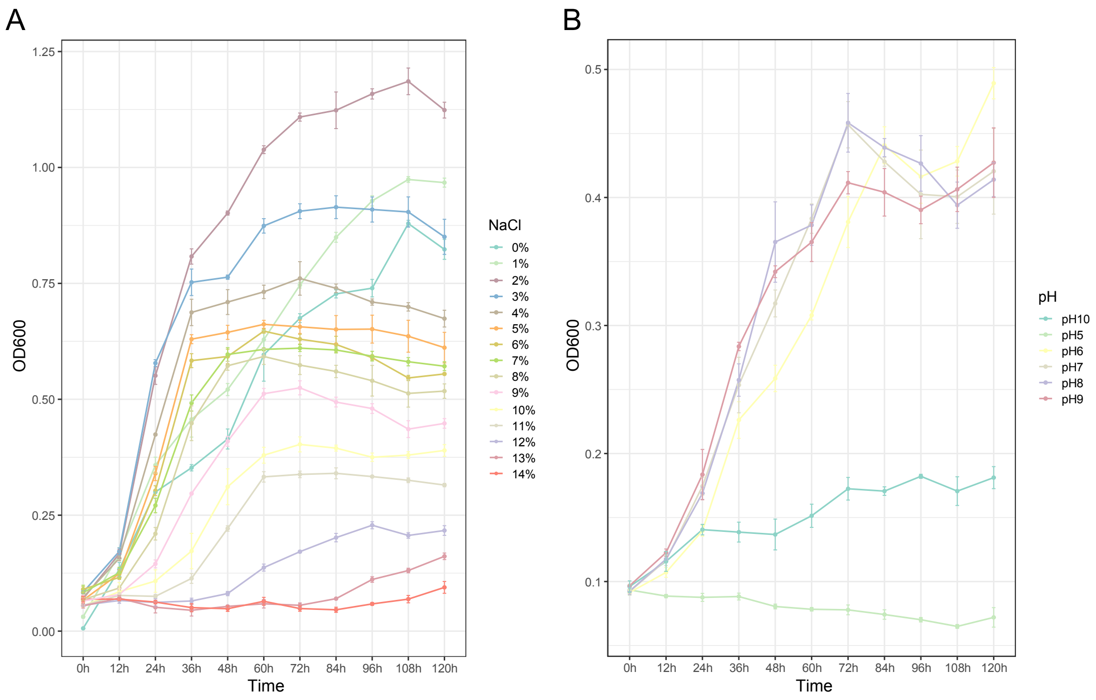

Fig S1. Growth curves of strain FT118^T in 2216 broth at 37°C under (A) different salinities and (B) pH. OD were measured in triplicates and error bars represented standard errors.

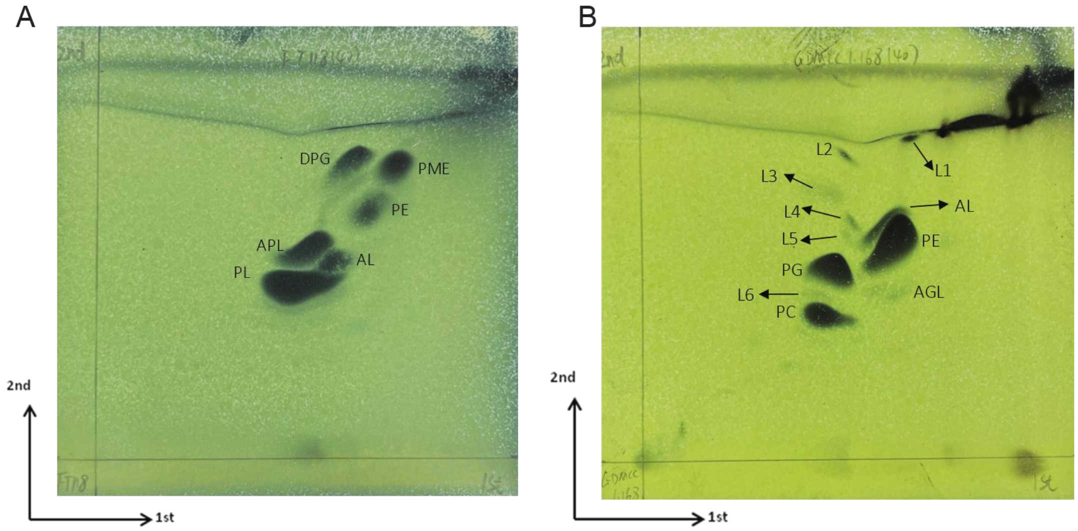

Fig S2. Two-dimentional TLC plates showing polar lipid profiles of (A) strain FT118^T and (B) *Rhodobacter capsulatus* GDMCC 1.168^T. DPG, diphosphatidylglycerol; PE, phosphatidylethanolamine; PME, phosphatidylmethylethanolamine; PG, phosphatidylglycerol; PC, phosphatidylcholine; AL, unidentified aminolipid; AGL, unknown aminoglycolipid; APL, unidentified aminophospholipid; PL, unidentified phospholipid; L1-6, unidentified lipids.

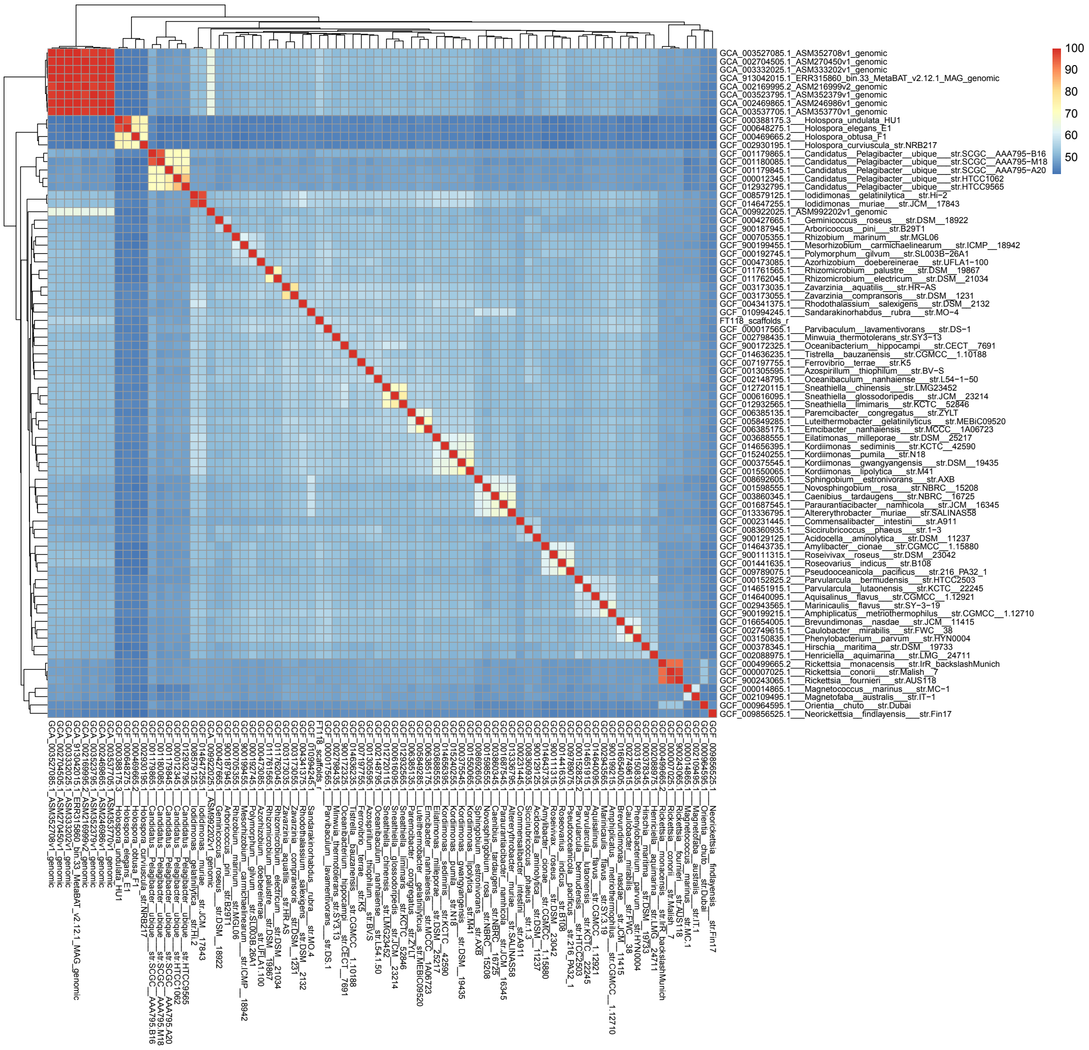

Fig S3. Pairwise AAI comparison between *Futianiales* genomes and representative genomes in *Alphaproteobacteria*. The result was obtained using CompareM v0.1.2 (https://github.com/dparks1134/CompareM). *Futianiales* genomes were marked in red.

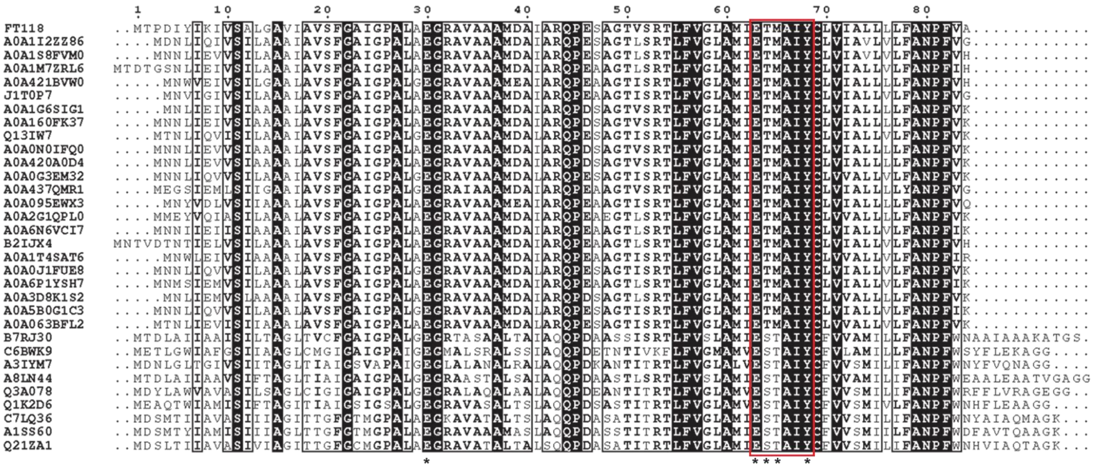

Fig S4. Multiple sequence alignment of c subunits of N-type ATPases in strain FT118^T and other *Pseudomonadota* representatives. Sequences are listed under their Uniprot accession numbers. Amino acid residues that function as Na^+/H^+-binding sites are marked with asterisks. The "ESTxxY" motif for Na^+ binding is boxed in red.

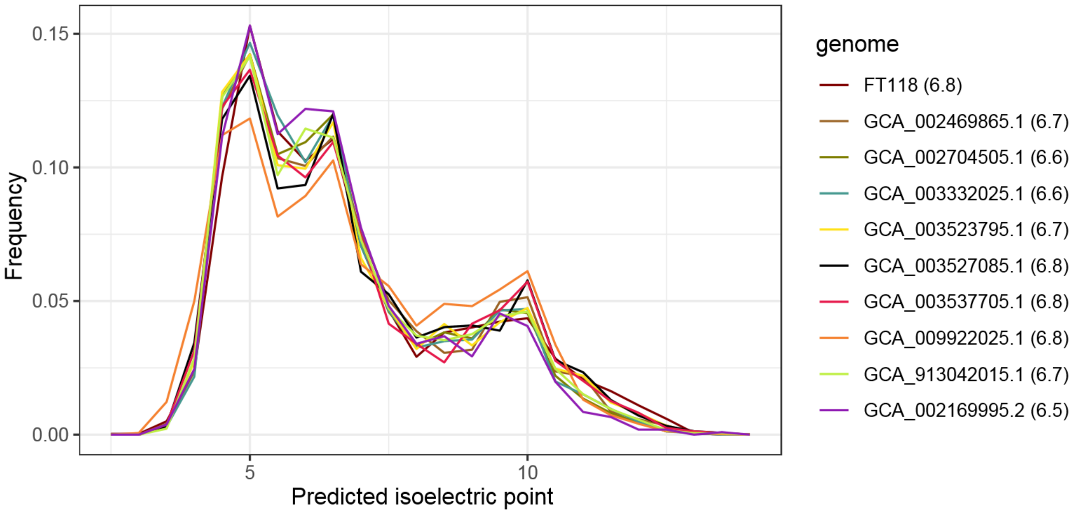

Fig S5. Isoelectric point profiles of *Futianiales* genomes. Numbers in the parentheses indicate the average isoelectric point of each genome.

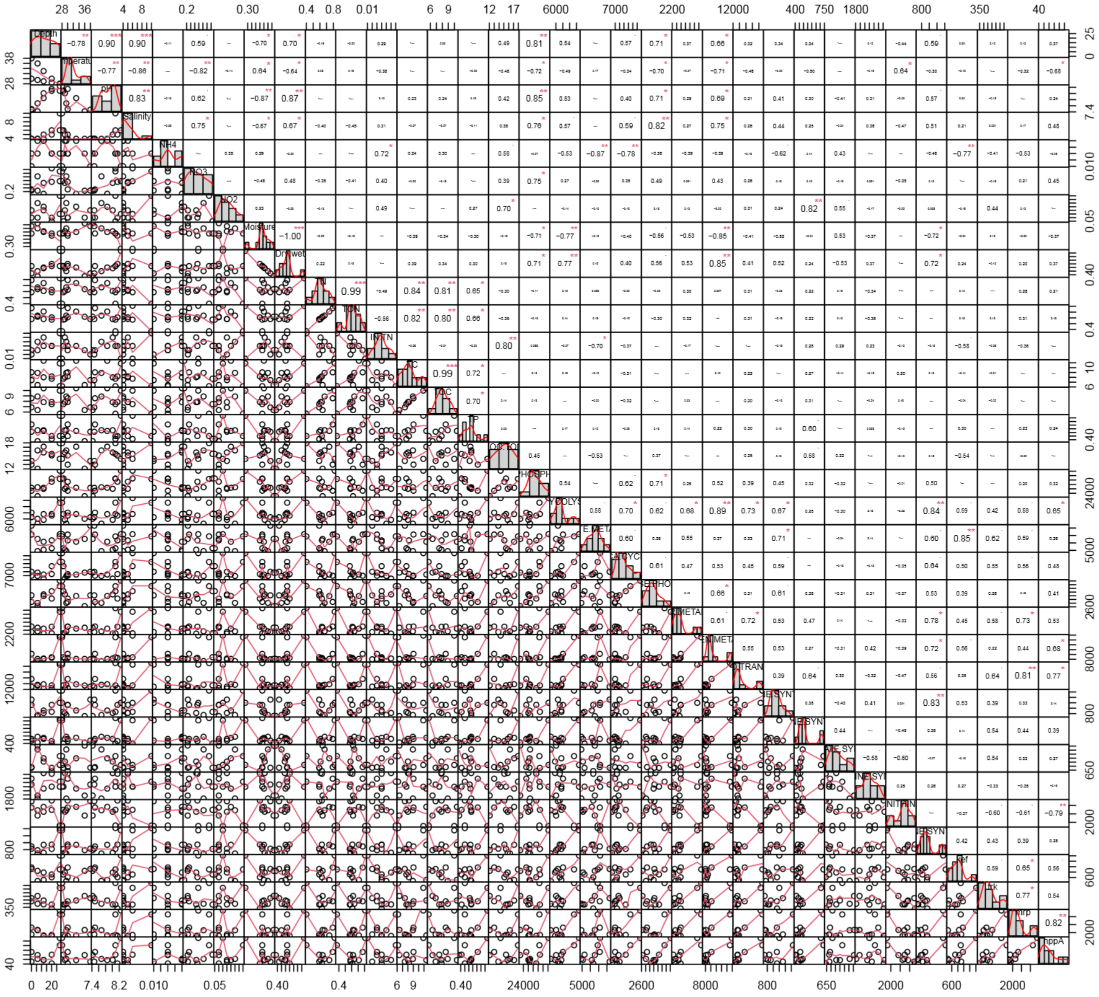

Fig S6. Spearman correlation between the expression level of major metabolic pathway/adaptive features in FT118^{T} and measured environmental variables of mangrove sediment samples. Data (from left to right) include depth, temperature, pH, salinity, NH_4^+-N, NO_3^--N, NO_2^--N, moisture, dry/wet ratio, total nitrogen, total organic nitrogen, inorganic nitrogen/total nitrogen, total carbon, total organic carbon, total phosphorus, total organic carbon/total organic nitrogen, the expression of oxidative phosphorylation, glycolysis, pyruvate metabolism, TCA cycle, pentose phosphate, sulfur metabolism, nitrogen metabolism, amino acid transporters (*aap*JQMP, *liv*FGHKM, ABC.PA.S), alanine synthesis (*dat*, *alr*, *ald*), ectoine/hydroxyectoine synthesis (*ect*ABCD), glutamate synthesis (*glt*BD), glutamine synthesis (*gln*A), N-∂-Acetyl-ornithine synthesis (*arg*BCDJ), proline synthesis (*pro*ABC), *kef*ABC, *trk*AH, Mrp complex and *hpp*A. The expression level of a pathway is represented by the sum of TPM values of all genes involved in the pathway. The TPM values of genes were listed in Table S4. The measured environmental variables of sediment samples were given in Table S7. The Spearman correlation analysis was performed using R package PerformanceAnalytics (1). The symbol "***", "**", "*", "." and " " corresponded with p<0.001, p<0.01, p<0.05, p<0.1 and p<1, respectively.

Table S1A. Complete results of API 20NE test of *Futiania mangrovii* FT118^T. +, positive; -, negative; w, weakly positive.

API 20NE Reaction/Substrate	FT118
Reduction of nitrate to nitrite	+
Denitrification	-
Indole production	-
D-glucose fermentation	-
Arginine DiHydrolase	-
UREase	-
β-glucosidase (Aesculin hydrolysis)	-
Gelatin hydrolysis	+
Beta-galactosidase	+
D-glucose	-
L-arabinose	-
D-mannose	-
D-mannitol	-
N-acetyl-glucosamine	-
D-maltose	-
Potassium gluconate	-
Capric acid	-
Adipic acid	-
Malic acid	-
Trisodium citrate	-
Phenylacetic acid	-

Table S1B. Complete results of API ZYM test of *Futiania mangrovii* FT118^{T}. +, positive; -, negative; w, weakly positive.

API ZYM Reaction/Substrate	FT118
Water	-
Alkaline phosphatase	+
Esterase(C4)	+
Esterase lipase (C8)	+
Lipase (C14)	-
Leucine arylamidase	+
Valine arylamidase	+
Cystine arylamidase	-
Trypsin	-
α-chymotrypsin	-
Acid phosphatase	w
Naphtol-AS-BI-phosphohydrolase	+
α-galactosidase	-
β-galactosidase	-
β-glucuronidase	-
α-glucosidase	-
β-glucosidase	-
N-acetyl-β-glucosaminidase	-
α-mannosidase	-
α-fucosidase	-

Table S1C. Complete results of Biolog GNIII test of *Futiania mangrovii* FT118^{T}. +, positive; -, negative; w, weakly positive.

Biolog GNIII substrate	Well	FT118	Biolog GNIII substrate	Well	FT118
Negative Control	A1	-	Gelatin	E1	-
Dextrin	A2	w	Glycyl-L-Proline	E2	-
D-Maltose	A3	-	L-Alanine	E3	-
D-Trehalose	A4	-	L-Arginine	E4	-
D-Cellobiose	A5	-	L-Aspartic Acid	E5	-
Gentiobiose	A6	-	L-Glutamic Acid	E6	-
Sucrose	A7	-	L-Histidine	E7	w
D-Turanose	A8	w	L-Pyroglutamic Acid	E8	-
Stachyose	A9	-	L-Serine	E9	-
Positive Control	A10	+	Lincomycin	E10	w
pH 6	A11	+	Guanidine HCl	E11	w
pH 5	A12	-	Niaproof 4	E12	w
D-Raffinose	B1	-	Pectin	F1	-
α-D-Lactose	B2	-	D-Galacturonic Acid	F2	w
D-Melibiose	B3	-	L-Galactonic Acid Lactone	F3	w
β-Methyl-D-Glucoside	B4	-	D-Gluconic Acid	F4	-
D-Salicin	B5	-	D-Glucuronic Acid	F5	w
N-Acetyl-D-Glucosamine	B6	-	Glucuronamide	F6	+
N-Acetyl-β-DMannosamine	B7	-	Mucic Acid	F7	-
N-Acetyl-D-Galactosamine	B8	-	Quinic Acid	F8	-
N-AcetylNeuraminic Acid	B9	+	D-Saccharic Acid	F9	w
1% NaCl	B10	w	Vancomycin	F10	w
4% NaCl	B11	w	Tetrazolium Violet	F11	w
8% NaCl	B12	w	Tetrazolium Blue	F12	w
α-D-Glucose	C1	-	p-Hydroxy-Phenylacetic Acid	G1	-
D-Mannose	C2	-	Methyl Pyruvate	G2	-
D-Fructose	C3	w	D-Lactic Acid Methyl Ester	G3	-
D-Galactose	C4	-	L-Lactic Acid	G4	-
3-Methyl Glucose	C5	-	Citric Acid	G5	-
D-Fucose	C6	w	α-Keto-Glutaric Acid	G6	w
L-Fucose	C7	w	D-Malic Acid	G7	-
L-Rhamnose	C8	w	L-Malic Acid	G8	w
Inosine	C9	-	Bromo-Succinic Acid	G9	-
1% Sodium Lactate	C10	w	Nalidixic Acid	G10	w
Fusidic Acid	C11	w	Lithium Chloride	G11	-
D-Serine	C12	w	Potassium Tellurite	G12	w
D-Sorbitol	D1	-	Tween 40	H1	-
D-Mannitol	D2	-	γ-Amino-Butryric Acid	H2	-

D-Arabitol	D3	-	α-Hydroxy-Butyric Acid	H3	-
myo-Inositol	D4	+	β-Hydroxy-D,LButyricAcid	H4	-
Glycerol	D5	-	α-Keto-Butyric Acid	H5	-
D-Glucose-6-PO4	D6	-	Acetoacetic Acid	H6	w
D-Fructose-6-PO4	D7	+	Propionic Acid	H7	-
D-Aspartic Acid	D8	-	Acetic Acid	H8	-
D-Serine	D9	w	Formic Acid	H9	-
Troleandomycin	D10	w	Aztreonam	H10	w
Rifamycin SV	D11	w	Sodium Butyrate	H11	w
Minocycline	D12	w	Sodium Bromate	H12	w

Table S2. Cellular fatty acid profile of strain FT118^T and representative species of related orders in *Alphaproteobacteria* (percent in total fatty acid). Major fatty acids (> 5%) were indicated in bold type. Components less than 0.1% were shown as trace. -, not detected. Data for *Rhodobacter capsulatus* GDMCC 1.168^T is obtained from this study. Data for *Caulobacter vibriodes* DSM 4738^T is obtained from (2-3). Data for *Parvularcula bermudensis* HTCC2503^T is obtained from (4). Data for *Micropepsis pineolensis* JCM 30711^T is obtained from (5).

		FT118^T	*Rhodobacter capsulatus* GDMCC 1.168^T	*Caulobacter vibrioides* DSM 4738^T	*Parvularcula bermudensis* HTCC2503^T	*Micropepsis pineolensis* JCM 30711^T
Saturated	C12:0	0.43	-	trace	**5.2**	0.4
	C13:0	0.23	-	-	-	-
	C14:0	0.59	0.12	1.1	2.9	**17**
	C15:0	-	-	1	-	0.2
	C16:0	**7.62**	1.81	**16.7**	**8.6**	**13.2**
	C17:0	1.62	0.11	trace	-	-
	C18:0	2.8	0.96	0.6	4	2.2
	C19:0 cyclo *ω*8*c*	**16.1**	-	-	**22**	-
Unsaturated	C17:1 *ω*6*c*	-	trace	-	-	-
	C17:1 *ω*8*c*	-	trace	-	-	-
	C18:1 *ω*5*c*	0.39	0.13	-	-	-
	C18:1 *ω*7*c*	**54.06**	**75.98**	**35.5**	**73.3**	**36.7**
	C18:1 *ω*7*c* 11-methyl	4.72	-	6.2	-	0.9
	C18:1 *ω*9*c*	-	-	trace	6	-
	C20:1 *ω*7*c*	0.56	-	-	-	-
	C20:2 *ω*6,9*c*	0.32	-	-	-	0.6
Branched chain	iso-C13:0 3OH	-	-	trace	-	-
	iso-C15:0	-	-	**8.9**	-	-
	C15:0 anteiso	-	0.14	trace	-	-
	iso-C16:0	-	-	0.5	-	-
	iso-C17:1 *ω*9*c*	-	-	2.3	-	-
	iso-C17:0	-	-	**11.2**	-	-
	C17:0 anteiso	-	0.09	1.9	-	-
	C18:0 iso	-	**7.59**	-	-	-
Hydroxy substituted	C10:0 3OH	-	2.16	-	-	-
	C12:0 3OH	-	-	trace	-	-
	C12:1 3OH	-	-	1.2	-	-
	C14:0 2OH	-	-	-	-	-
	C15:0 3OH	-	-	-	-	0.1
	C16:0 3OH	0.53	0.11	-	-	4.6
	C17:0 3OH	0.51	-	-	-	-

C18:0 3OH	3.45	1.61	-	-	-
C18:1 2OH	4.09	-	-	-	-
Summed feature 2*	0.94	-	-	-	-
Summed feature 3*	0.46	**8.43**	**9**	-	0.8
Summed feature 7*	0.58	0.76	-	-	0.2

*Summed Features are fatty acids that cannot be resolved reliably from another fatty acid using the chromatographic conditions chosen. The MIDI system groups these fatty acids together as one feature with a single percentage of the total. Summed feature 2 contained C14:0 3-OH and/or iso I-C16:1, Summed Feature 3 contained C16:1 *ω*7*c* and/or C16:1 *ω*6*c*, Summed Feature 7 contained C19:1 *ω*6*c*, C19:1 *ω*7*c* and/or C19:0 cyclo *ω*10*c*.

Table S3. Genomic features of *Futianiales* genomes.

Genome	Genbank accession	Size (bp)	GC content (mol%)	#Contig	Largest contig (bp)	N50 (bp)	Completeness (%)	Contamination (%)	Reference
FT118	JAMZFT000000000	3397356	68.5	6	1448022	1219768	99.57	0.22	This study
Rhodobacteraceae bacterium	GCA_002704505.1	2165014	50.8	32	375574	119749	94.66	8.8	-
Alphaproteobacteria bacterium	GCA_009922025.1	4117200	45.6	971	78196	10111	94.08	9.93	(6)
Alphaproteobacteria bacterium UBA7371	GCA_002469865.1	1629558	49.9	175	37017	11712	83.8	0.88	(7)
SAR116 cluster bacterium	GCA_003332025.1	1613672	51.2	42	111444	57987	82.88	0	-
uncultured Alphaproteobacteria bacterium	GCA_913042015.1	1585126	51.1	230	30590	8473	82.82	1.2	-
Alphaproteobacteria bacterium	GCA_003537705.1	1385405	50	261	22506	5615	66.68	0.93	-
Alphaproteobacteria bacterium	GCA_003523795.1	1280707	50	252	15724	5408	63.16	1.2	-
Alphaproteobacteria bacterium	GCA_003527085.1	1343714	49.6	263	14912	5452	61.1	0	-
Alphaproteobacteria bacterium TMED150	GCA_002169995.2	1038806	50.6	5	375574	223112	60.34	0	(6)

Table S4. Relative expression level (TPM) of genes involved in essential metabolic pathways in strain FT118^{T}.

Gene name	EC	KO	MF1 0-2cm	MF1 6-8cm	MF1 12-14cm	MF1 20-22cm	MF1 28-30cm	MG1 0-2cm	MG1 6-8cm	MG1 12-14cm	MG1 20-22cm	MG1 28-30cm
OXIDATIVE PHOSPHORYLATION												
nuoA	1.6.5.3	ko:K00330	1663.93	1289.45	1668.24	1884.24	2329.13	1351.23	1562.06	1636.75	1879.96	1820.29
nuoB	1.6.5.3	ko:K00331	211.44	202.45	180.42	211.00	319.27	200.56	228.67	177.40	231.53	342.92
nuoC	1.6.5.3	ko:K00332	743.98	629.71	744.10	851.95	1054.17	572.10	766.52	748.69	948.85	842.57
nuoD	1.6.5.3	ko:K00333	151.91	159.19	173.55	161.93	188.41	193.58	223.28	164.12	91.52	221.93
nuoE	1.6.5.3,1.6.99.3	ko:K00334,ko:K03943	637.78	550.03	665.56	692.74	1005.86	635.46	558.45	705.86	780.53	937.74
nuoF	1.6.5.3	ko:K00335	651.78	608.05	697.49	747.11	966.94	622.34	714.55	822.94	839.55	819.12
nuoG	1.6.5.3	ko:K00336	256.05	229.63	263.13	322.72	527.08	318.48	305.67	299.21	300.91	475.22
nuoH	1.6.5.3	ko:K00337	1191.96	1139.10	1454.97	1621.51	2037.08	1042.77	1397.48	1748.39	1566.17	1580.84
nuoI	1.6.5.3,1.6.99.3	ko:K00338,ko:K03941	241.38	254.61	225.33	238.78	463.26	220.28	221.25	213.78	313.13	551.12
nuoJ	1.6.5.3	ko:K00339	261.81	310.33	442.73	501.39	706.74	314.22	420.27	413.55	624.61	559.85
nuoK	1.6.5.3	ko:K00340	357.66	383.28	458.40	526.67	1016.27	455.65	491.76	533.62	601.58	823.26
nuoL	1.6.5.3	ko:K00341	335.97	410.15	478.31	516.92	1032.94	420.48	565.68	560.13	614.18	832.01
nuoM	1.6.5.3	ko:K00342	181.51	187.33	192.71	200.98	362.69	215.30	236.89	169.03	222.41	325.87
nuoN	1.6.5.3	ko:K00343	181.27	363.23	286.99	160.11	183.82	296.34	438.77	427.34	195.55	207.24
sdhC	-	ko:K00241	151.79	139.55	113.03	148.00	198.30	161.90	189.71	76.59	169.32	344.25
sdhD	-	ko:K00242	753.85	556.87	609.13	688.18	1374.58	622.19	511.65	549.56	693.90	1154.09
sdhA	1.3.5.1,1.3.5.4	ko:K00239	623.12	449.35	600.63	586.69	965.77	638.74	539.02	527.17	757.84	896.95
sdhB	1.3.5.1,1.3.5.4	ko:K00240	32.68	28.66	17.87	44.00	81.77	17.04	64.38	0.00	0.00	81.82
petC	-	ko:K00413	166.88	166.41	184.47	139.10	209.74	131.72	175.32	111.04	148.21	227.00
petB	-	ko:K00410,ko:K00412	366.58	277.16	314.01	419.12	280.49	257.00	309.39	321.32	389.02	320.33
petA	1.10.2.2	ko:K00411	284.73	201.63	221.69	233.83	329.36	247.54	262.56	190.38	218.53	332.13
coxC	1.9.3.1	ko:K02276	386.94	365.41	451.82	454.33	240.04	755.45	777.40	468.85	488.75	207.81
coxA	1.9.3.1	ko:K02274	1156.10	1024.78	1281.95	1525.31	985.68	1139.29	1358.38	1474.67	1755.33	886.60

coxB	1.9.3.1	ko:K02275	107.22	104.91	122.94	143.58	144.17	125.68	88.59	86.56	119.20	210.83
ccoN	1.9.3.1	ko:K00404	708.62	530.96	738.47	773.33	743.89	794.67	777.96	662.44	917.17	626.97
ccoO	-	ko:K00405	67.00	68.37	113.21	86.11	104.49	50.80	105.58	73.76	33.18	52.75
ccoQ	-	ko:K00407	110.89	126.43	161.66	128.31	194.32	148.53	172.93	121.24	168.35	150.70
ccoP	-	ko:K00406	243.17	219.33	189.97	192.73	288.85	265.77	190.31	223.83	221.41	239.05
atpG	-	ko:K02115	284.43	242.72	297.01	300.14	252.52	221.83	174.69	216.36	201.59	286.18
atpA_1	3.4.21.68 ,3.6.3.14	ko:K01343,ko:K02111	229.55	244.39	234.21	232.71	425.49	270.87	208.22	187.21	262.22	371.70
atpF	-	ko:K02109	300.42	240.62	309.59	189.72	382.62	284.58	253.48	214.64	344.02	437.51
atpE	-	-	185.23	226.73	180.98	159.19	338.05	254.75	167.69	119.32	214.72	384.00
atpB_1	-	ko:K02108	185.70	352.54	189.51	139.23	243.62	384.69	319.04	326.31	192.43	264.91
atpR	-	-	120.16	71.53	111.47	107.86	208.22	151.85	97.55	88.19	104.15	165.57
atpQ	-	ko:K02116	310.04	310.04	148.07	225.52	529.86	213.02	275.18	93.51	165.04	451.41
atpC_2	-	ko:K02114	256.82	401.12	268.60	425.48	411.86	310.45	343.13	234.39	246.39	338.49
atpD_1	3.6.3.14	ko:K02112	285.11	244.20	307.23	288.96	473.34	310.77	283.46	280.45	341.04	418.90
atpG_1	-	ko:K02115	261.77	297.76	334.04	274.81	563.52	327.87	291.52	252.40	354.34	516.30
atpA_2	3.6.3.14	ko:K02111	310.96	346.17	278.96	268.35	381.15	351.06	285.43	210.96	252.01	489.18
-	-	ko:K02109	140.48	181.96	232.37	143.07	482.34	177.76	175.97	134.50	171.72	372.42
-	-	ko:K02110	114.35	115.15	116.21	154.87	321.20	148.68	170.67	90.96	113.61	310.96
atpB_1	-	ko:K02108	131.15	108.65	89.84	93.61	341.86	123.01	89.64	70.16	68.87	379.78
glcD	1.1.3.15	ko:K00104	232.53	261.51	247.99	258.64	703.43	240.33	229.43	233.31	264.40	495.84
-	-	ko:K02116	120.90	144.91	179.00	118.69	244.86	115.54	190.52	96.59	92.63	316.31
atpC_1	-	ko:K02114	228.49	264.22	333.89	248.31	419.76	320.09	323.96	292.51	305.46	408.86
-	3.6.3.14	ko:K02112	145.12	151.03	133.55	117.24	225.91	173.16	195.34	122.04	146.60	185.92
atpH	-	ko:K02113	4264.45	2616.19	3617.83	4398.47	3172.70	3292.61	3428.76	3444.94	4527.42	2515.58
atpA	3.6.3.14	ko:K02111	280.29	219.84	284.05	362.06	317.10	195.62	264.85	257.57	266.94	242.40
atpG	-	ko:K02115	4542.71	3282.98	4203.64	5268.56	3724.08	3651.40	3887.57	4312.98	5541.59	3016.28
atpD	3.6.3.14	ko:K02112	95.71	120.17	119.80	140.74	164.44	138.30	146.86	88.96	128.65	204.51

atpC	-	ko:K02113,ko:K02114	137.43	141.19	129.47	107.39	176.01	133.05	116.02	116.40	135.38	118.23
atpI	-	ko:K02116	101.07	105.36	72.62	60.27	163.77	68.25	91.02	58.29	90.47	131.31
atpB	-	ko:K02108	126.14	122.92	124.13	133.05	140.93	147.11	113.75	91.94	131.73	121.40
atpE	-	ko:K02110	519.65	551.41	523.58	505.97	665.15	623.89	609.12	455.09	438.73	1036.87
atpF2	-	ko:K02109	226.02	247.66	237.29	181.87	258.34	197.79	204.35	308.98	181.53	314.90
atpF2	-	ko:K02109	92.59	105.11	125.12	126.07	152.70	104.13	127.88	111.29	124.70	138.54
GLYCOLYSIS											
algC	5.4.2.2,5.4.2.8	ko:K15778	73.81	89.91	82.48	95.01	122.14	100.35	103.53	60.57	76.23	126.45
pgi	5.3.1.9	ko:K01810	142.19	201.15	208.28	152.75	259.73	207.80	167.31	146.98	181.15	282.17
pfkB	2.7.1.11	ko:K16370	92.50	159.61	164.00	124.54	328.80	227.63	98.49	171.55	170.25	292.30
glpX	3.1.3.11, 3.1.3.37	ko:K02446,ko:K11532	306.39	256.81	298.82	287.67	404.54	292.47	251.07	256.43	331.08	408.67
fbaB	4.1.2.13	ko:K01623	199.51	235.03	215.45	180.41	342.75	280.09	199.52	259.51	228.81	265.60
gapA	1.2.1.12	ko:K00134	927.18	797.42	957.65	943.68	844.27	910.56	893.97	874.34	1017.79	779.85
pgk	2.7.2.3	ko:K00927	211.26	212.00	222.25	231.75	341.74	246.88	194.98	199.61	202.92	325.77
gpmI	5.4.2.12	ko:K15633	95.56	168.74	99.79	75.17	143.26	116.42	101.52	104.02	61.42	116.24
eno	4.2.1.11	ko:K01689	1059.59	879.27	1000.10	1144.38	651.57	748.88	623.37	857.15	739.43	633.39
pyk	2.7.1.40	ko:K00873	270.43	268.32	292.25	208.13	453.30	250.79	198.51	220.45	224.81	494.89
ppdK	2.7.9.1	ko:K01006	1125.12	1171.19	1245.41	1218.86	1381.07	1300.71	1271.22	1378.41	1429.95	1155.71
pdhA	1.2.4.1	ko:K00161	118.77	76.92	66.59	147.61	211.88	105.84	76.79	61.47	41.48	235.74
pdhC	1.2.4.1,2.3.1.12	ko:K00162,ko:K00627	407.33	412.19	433.12	412.56	504.77	382.20	338.63	361.43	510.48	453.14
pdhB	1.2.4.1	ko:K00162	423.54	301.22	337.85	415.39	296.80	306.44	257.88	288.52	399.24	301.18
-	1.2.4.1	ko:K00162,ko:K21417	232.23	250.17	242.68	194.39	553.83	402.82	323.06	191.85	251.93	654.14
MA20_17010	1.2.4.1	ko:K00163	163.40	228.13	202.39	166.52	320.83	246.50	236.85	167.79	162.32	282.87
-	2.3.1.12	ko:K00627	154.41	178.11	196.73	135.66	270.63	216.47	154.66	158.47	206.75	273.42
pckA	4.1.1.49	ko:K01610	143.04	128.52	129.67	143.47	252.50	170.44	133.99	176.03	163.03	238.62
PYRUVATE METABOLISM											

phbC	2.3.1.304	ko:K03821	821.01	677.69	694.34	877.17	480.87	632.81	578.10	742.50	725.00	502.84
phbB	1.1.1.36	ko:K00023	2256.00	1718.35	1843.04	1894.46	1328.00	1370.90	1071.29	1310.00	1301.90	1141.67
phbA	2.3.1.9	ko:K00626	161.74	163.60	135.56	96.18	245.32	205.45	137.15	102.74	98.41	281.78
phaZ	3.1.1.75	ko:K05973	100.66	93.78	101.04	118.87	129.75	128.46	80.62	49.96	64.06	157.22
maeA	1.1.1.38	ko:K00027	220.44	269.15	228.62	227.70	373.86	205.80	182.21	186.48	229.61	422.34
maeB	1.1.1.40	ko:K00029	713.76	637.22	796.94	684.55	1122.42	638.30	559.59	674.10	687.71	984.86
acs	6.2.1.1	ko:K01895	2128.23	1960.35	1548.44	1494.97	2031.82	2614.56	2116.31	1784.82	1797.10	2148.05
aldH	1.2.1.3	ko:K00128	257.56	246.07	232.62	278.51	375.21	269.23	227.06	200.19	240.45	300.80
TCA CYCLE												
mdh	1.1.1.37	ko:K00024	1589.88	1345.69	1523.31	1465.86	1765.42	1303.30	1262.22	1330.58	1327.88	1573.43
fumC	4.2.1.2	ko:K01679	289.96	318.93	332.40	301.17	605.25	297.93	238.41	266.09	265.92	511.25
sdhA	1.3.5.1,1.3.5.4	ko:K00239	623.12	449.35	600.63	586.69	965.77	638.74	539.02	527.17	757.84	896.95
sdhB	1.3.5.1,1.3.5.4	ko:K00240	32.68	28.66	17.87	44.00	81.77	17.04	64.38	0.00	0.00	81.82
sucD	6.2.1.5	ko:K01902	497.84	445.52	502.17	538.98	688.06	447.33	435.23	446.37	523.76	609.71
sucC	6.2.1.5	ko:K01903	2488.27	2781.71	2198.26	2407.32	2920.48	2229.51	2381.88	2186.99	3254.09	2374.13
sucA	1.2.4.2	ko:K00164	496.78	415.29	417.83	493.18	585.85	441.38	437.57	405.35	604.12	667.68
sucB	2.3.1.61	ko:K00658	364.56	442.05	399.78	343.55	492.84	434.47	485.78	335.91	404.82	500.02
idh1	1.1.1.42	ko:K00031	454.38	374.65	379.42	461.64	486.81	375.77	359.34	420.07	413.92	368.95
acnA	4.2.1.3	ko:K01681	248.30	326.77	305.23	179.03	403.99	291.05	327.62	275.79	234.14	360.28
gltA	2.3.3.1	ko:K01647	408.28	458.05	468.33	379.82	829.31	511.97	423.78	435.84	404.44	777.45
PENTOSE PHOSPHATE												
cbbT	2.2.1.1	ko:K00615	482.96	481.08	527.52	504.60	891.41	516.40	559.45	582.53	657.84	748.11
rpe	5.1.3.1	ko:K01783	581.42	379.98	496.16	507.66	606.90	481.06	411.62	588.26	540.70	645.46
rpiA	5.3.1.6	ko:K01807	662.05	489.36	573.47	570.51	915.21	646.88	513.27	460.24	675.51	906.30
prs	2.7.6.1	ko:K00948	1285.53	1052.72	1329.06	1232.01	1224.74	974.03	1108.42	1417.48	1365.16	1076.35
pmm-pgm	5.4.2.2,5.4.2.8	ko:K15778	73.81	89.91	82.48	95.01	122.14	100.35	103.53	60.57	76.23	126.45

phnN	2.7.4.23	ko:K05774	139.58	149.34	177.41	139.77	260.24	147.95	118.23	165.51	133.08	217.22
SULFUR METABOLISM												
sulP	-	ko:K03321	573.31	550.93	563.45	744.74	511.32	569.26	543.19	588.59	673.17	545.71
cysD	2.7.7.4	ko:K00957	161.80	163.19	194.88	154.03	188.55	192.42	119.52	136.50	154.10	260.03
cysNC	2.7.1.25, 2.7.7.4	ko:K00955	244.19	248.57	291.25	308.07	280.98	302.47	334.25	268.03	281.80	326.23
cysH	1.8.4.10, 1.8.4.8	ko:K00390	228.23	242.78	229.42	208.17	178.93	184.22	98.62	104.02	85.24	322.82
cysI	1.8.1.2	ko:K00381	168.08	192.67	200.61	159.38	318.15	243.53	152.96	179.19	167.68	334.48
cysK	2.5.1.47	ko:K01738	209.08	265.00	232.30	216.44	343.92	195.33	253.32	181.51	150.05	324.94
cysB	2.5.1.47	ko:K01738	104.86	150.10	131.58	132.66	311.22	161.42	153.05	159.09	131.32	285.72
metY	2.5.1.47, 2.5.1.49	ko:K01738,ko:K01740	63.11	34.06	21.23	32.68	64.78	121.48	53.56	29.40	13.23	68.33
metB	2.5.1.48	ko:K01739,ko:K10764	190.95	180.75	203.83	156.26	245.93	175.63	181.59	168.62	152.47	204.50
tauE	-	ko:K07090	366.92	500.96	413.86	465.04	882.46	441.53	455.18	418.83	438.23	834.56
NITROGEN METABOLISM												
narK	-	ko:K02575	336.59	242.73	223.98	260.31	593.37	507.37	241.58	274.29	341.39	607.64
narG	1.7.5.1	ko:K00370	426.64	310.90	325.53	308.98	849.41	726.47	329.42	358.03	460.32	742.48
narH	1.7.5.1	ko:K00371	417.66	308.04	213.53	162.18	243.19	568.72	232.50	197.08	319.39	241.52
narI	1.7.5.1	ko:K00374	199.28	214.44	165.76	158.03	360.06	242.19	179.18	103.64	164.86	393.78
amt	-	ko:K03320	348.52	316.11	353.24	300.33	489.69	321.87	324.45	306.66	359.41	455.64
urtA	-	ko:K11959	211.22	258.97	229.60	610.15	539.81	272.51	263.95	272.96	391.64	501.02
urtB	-	ko:K11960	161.40	262.50	222.14	205.60	743.87	238.89	229.67	176.50	198.51	679.65
urtC	-	ko:K11961	316.30	275.29	284.55	308.18	690.70	399.81	341.77	282.84	269.95	499.94
urtD	-	ko:K11962	235.78	157.36	195.71	232.29	537.15	212.85	192.58	206.03	216.39	444.39
urtE	-	ko:K11963	169.84	194.62	160.00	113.95	298.55	176.47	163.00	168.44	136.08	284.41
ureA	3.5.1.5	ko:K01430,ko:K14048	380.25	381.13	311.83	231.61	431.45	302.06	285.35	374.61	302.13	548.91
ureB	3.5.1.5	ko:K01429	362.31	349.66	434.71	417.01	1310.70	442.39	312.11	384.92	460.28	1025.02
ureC	3.5.1.5	ko:K01428	129.51	187.20	113.93	100.96	236.67	129.96	173.41	133.65	225.49	314.17

ureD	-	ko:K03190	233.86	294.89	283.34	321.26	712.34	294.34	234.07	264.71	342.21	771.53
ureE	-	ko:K03187	151.30	167.88	133.89	125.08	264.94	129.14	168.57	154.83	168.71	268.19
ureF	-	ko:K03188	653.53	868.73	1076.56	891.53	910.83	749.65	830.09	898.87	815.54	888.04
ureG	-	ko:K03189	116.47	118.29	118.86	92.60	143.78	117.96	87.40	85.08	63.46	139.90
glnA	6.3.1.2	ko:K01915	2152.62	1884.52	2052.98	2412.65	2098.73	2293.87	1813.02	2430.57	2100.89	2220.85
gltB	1.4.1.13, 1.4.1.14	ko:K00265	549.93	762.52	648.31	531.61	494.64	504.88	794.02	612.96	542.00	566.72
gltD	1.4.1.13, 1.4.1.14	ko:K00266	130.97	194.38	150.78	108.52	220.85	183.82	114.11	144.88	137.23	259.05
gdh2	1.4.1.2	ko:K15371	322.86	328.59	381.62	404.21	731.29	310.45	325.29	340.00	416.73	704.62
			#N/A	#N/A	#N/A	#N/A	#N/A	#N/A	#N/A	#N/A	#N/A	#N/A
CATION TRANSPORTERS												
pstS	-	ko:K02040	708.65	507.85	615.81	726.01	356.15	488.82	347.30	457.67	470.95	392.89
pstB	3.6.3.27	ko:K02036	602.27	657.59	613.22	659.27	2004.64	510.30	584.92	566.58	627.98	1587.16
pstA	-	ko:K02038	244.79	276.12	219.42	217.63	593.89	240.44	265.80	220.65	245.69	485.67
pstC	-	ko:K02037	213.73	198.24	219.36	182.37	549.71	242.96	197.39	177.85	207.80	478.96
znuB	-	ko:K09816	291.26	267.86	300.96	294.33	446.63	414.32	341.58	295.81	256.66	434.01
znuC	-	ko:K09817	216.86	221.01	242.90	146.39	360.72	234.47	286.18	187.42	180.91	391.30
znuA	-	ko:K09815	151.76	169.51	163.71	142.83	252.03	154.52	157.57	157.76	171.45	275.85
afuC	3.6.3.30	ko:K02010	117.49	168.38	156.49	170.82	274.00	157.25	186.14	133.71	147.27	210.43
afuB	-	ko:K02011	132.31	127.72	134.81	108.45	203.70	143.10	106.77	147.32	160.01	226.35
afuA	-	ko:K02012	271.18	159.88	204.31	3629.76	256.29	492.60	147.24	158.68	114.84	250.42
modC	3.6.3.29	ko:K02017	234.13	284.31	237.78	232.19	414.97	309.83	236.99	206.29	212.81	500.62
modA	-	ko:K02020	228.58	441.16	335.50	171.91	340.86	301.54	742.09	597.15	319.77	337.40
modB	-	ko:K02018	258.58	260.80	272.29	161.23	583.93	316.58	246.58	188.31	232.14	503.44
AMINO ACID TRANSPORTERS												
aapJ	-	ko:K09969	523.21	368.92	540.90	476.17	333.11	549.30	414.31	529.60	572.82	340.21
aapQ	-	ko:K09970	80.55	109.44	95.99	59.68	169.79	113.16	98.73	96.34	80.11	173.95
aapM	-	ko:K09971	358.27	288.57	324.55	350.29	269.68	553.85	382.65	337.06	442.10	306.34

aapP	3.6.3.21	294.04308	294.04	326.51	318.94	265.39	472.46	333.15	276.28	245.18	308.21	
livK	-	ko:K01999	1804.40	1758.04	1871.54	1751.35	2272.25	2283.23	1924.65	1580.48	1692.67	2364.16
livH	-	ko:K01997	2403.54	2299.66	2496.02	2116.92	3309.98	3178.05	2483.24	2134.48	2262.17	3528.17
livM	-	ko:K01998	1390.87	1600.97	1543.83	1518.96	2318.26	1513.61	1674.03	1534.76	1608.25	2410.49
livG	-	3042.5271	3042.53	3202.50	3175.79	2915.56	5762.20	3289.63	3325.68	2876.25	3280.91	
livF	-	ko:K01996	2545.09	2817.73	2898.32	2524.77	5010.06	3057.55	2516.52	2493.49	2668.78	4809.97
ABC.PA.S	3.6.3.21	ko:K02028,ko:K02029,ko:K02030,ko:K09972	194.90	215.80	181.47	135.31	215.51	194.74	143.97	110.64	167.99	250.57
ABC.PA.S	-	ko:K02029,ko:K02030	203.43	234.13	204.89	235.02	261.17	345.24	220.46	246.10	204.54	278.60
ABC.PA.S	-	ko:K02030	60.44	77.62	72.81	63.45	95.68	100.40	75.88	64.10	64.19	129.76
ABC.PA.S	-	ko:K02030,ko:K02424	71.69	74.58	74.34	71.57	83.06	104.82	79.87	72.35	72.40	113.08
HOMEOSTASIS												
kefA	-	ko:K05802	588.82	532.83	470.92	544.88	634.58	565.46	483.64	572.19	522.17	513.03
kefBC	-	ko:K03455,ko:K11745,ko:K11747	89.84	128.20	106.55	57.40	168.34	127.00	83.98	73.76	105.77	161.55
trkH	-	ko:K03498	237.92	257.11	206.13	158.30	243.82	170.25	166.54	175.38	185.89	300.44
trkA	-	ko:K03499	237.88	248.20	237.60	175.94	372.60	261.94	232.09	214.20	202.08	382.21
mrpA	-	ko:K05566	135.47	209.11	123.89	164.16	367.40	148.95	137.80	156.60	140.91	312.00
mrpB	-	ko:K05566	339.61	402.38	270.19	214.40	303.85	479.00	357.66	260.05	213.74	271.82
mrpC	-	ko:K05567	158.93	177.83	156.55	145.73	452.14	231.41	172.19	121.68	182.42	348.95
mrpD	-	ko:K05568	591.14	542.26	479.98	553.48	924.95	587.42	529.63	493.84	592.71	766.23
mrpE	-	ko:K05569	120.81	127.15	135.10	129.40	234.22	143.14	164.43	121.92	149.60	217.01
mrpD	1.6.5.3	294.8986	294.90	404.57	393.66	286.04	496.38	428.66	510.75	410.88	359.17	
mrpF	-	ko:K05570	94.82	170.36	118.21	169.44	222.98	173.70	129.88	112.69	112.26	285.92
mrpG	-	ko:K05571	157.51	135.41	150.39	140.39	386.93	124.90	148.58	116.42	108.78	409.51
hppA	3.6.1.1	ko:K15987	50.40	79.04	57.26	42.58	153.52	92.72	62.29	57.84	80.76	121.96

AMINO ACID SYNTHESIS												
dat	2.6.1.21	ko:K00824	135.74	148.49	110.52	113.50	221.89	147.11	135.14	109.74	152.59	250.87
alr	5.1.1.1	ko:K01775	227.21	196.17	263.39	216.53	278.25	226.34	256.06	223.58	214.96	391.93
ald	1.4.1.1	ko:K00259	696.53	411.30	484.42	632.30	499.94	586.48	383.86	478.92	466.09	583.49
ectA	2.3.1.178	ko:K06718	176.33	179.51	233.12	185.69	317.08	189.05	200.38	194.62	190.99	296.13
ectB	2.6.1.19, 2.6.1.76	ko:K00823,ko:K00836	61.37	44.85	43.34	65.42	77.99	49.77	78.58	77.42	26.12	91.36
ectC	4.2.1.108	ko:K06720	122.74	108.00	115.66	93.59	209.73	114.53	115.41	86.23	99.75	214.01
ectD	1.14.11.5 5	ko:K10674	78.05	80.62	83.57	87.58	103.84	107.00	95.67	102.84	82.69	137.32
gltB	1.4.1.13, 1.4.1.14	ko:K00265	549.93	762.52	648.31	531.61	494.64	504.88	794.02	612.96	542.00	566.72
gltD	1.4.1.13, 1.4.1.14	ko:K00266	130.97	194.38	150.78	108.52	220.85	183.82	114.11	144.88	137.23	259.05
glnA	6.3.1.2	ko:K01915	2152.62	1884.52	2052.98	2412.65	2098.73	2293.87	1813.02	2430.57	2100.89	2220.85
argJ	2.3.1.1,2. 3.1.35	ko:K00620	436.93	378.09	380.21	368.18	399.09	495.03	422.89	390.24	407.67	476.60
argD	2.6.1.11, 2.6.1.17	ko:K00821	341.63	372.66	345.82	414.66	543.48	363.99	305.87	338.06	424.57	521.51
argB	2.7.2.8	ko:K00930	407.22	434.77	414.83	393.39	495.74	525.93	408.15	386.67	385.57	488.39
argC	1.2.1.38	ko:K00145	2054.20	1217.60	1769.92	2105.31	352.23	1490.17	1576.89	1507.11	1520.43	333.42
proA	1.2.1.41	ko:K00147	212.36	200.12	229.28	275.17	348.07	266.19	200.94	181.14	227.53	338.14
proB	2.7.2.11	ko:K00931	480.55	400.01	489.72	500.66	685.24	407.95	324.97	368.20	429.59	680.89
proC	1.5.1.2	ko:K00286	135.73	184.78	221.03	171.86	324.23	219.19	164.74	163.20	164.42	403.32

Table S5. Sample information of 16S rRNA gene amplicon datasets in which *Futianiales* was present.

SRA accession	Bioproject	Biosample	Env_biome	Env_feature	Env_material	Env_package	Sample type	Latitude	Longitude	Relative_abundance
SRR1948179	PRJNA279913	SAMN03449765	bohai sea	marine sediment	original soil sample added Sodium benzoate		marine sediment	37.6	120.38	18.7611%
SRR1133270	PRJNA232457	SAMN02586139	coastal marsh		sediment		coastal sediment	29.17407	-90.64935	18.1571%
SRR1948178	PRJNA279913	SAMN03449764	bohai sea	marine sediment	original soil sample added Sodium benzoate		marine sediment	37.6	120.37	16.9267%
SRR5178048	PRJNA361424	SAMN06233416	marine	-	sediment	marine metagenome	marine sediment	21.18	72.1	7.9378%
SRR1066770	PRJNA229799	SAMN02427080	coastal saline soils Odisha	16S rRNA gene sequence	16S rRNA gene sequence	MIGS/MIMS/MIMARKS.soil	soil	19.416667	85.066667	7.0822%
ERR361767	PRJEB4793	SAMEA2242129	a sewage plant	a sewage plant for wastewater from oil reservoir	wasterwater	wastewater/sludge	wastewater/sludge	21.0333	109.1	6.9061%
SRR5468970	PRJNA381927	SAMN06820513	ENVO:01000022	ENVO:01001305	ENVO:00002243		soil	29.4406	-89.88492	6.4543%
ERR713048	PRJEB8105	SAMEA3182221	wastewater treatment plant	wastewater treatment plant	activated sludge	wastewater/sludge	wastewater/sludge	55.50727	12.14549	5.6226%
SRR8359316	PRJNA510850	SAMN10617124			activated sludge		wastewater/sludge	36.070613	120.42273	4.8457%
SRR3289600	PRJNA316054	SAMN04577806	marine	-	sediment		marine sediment	21.4	117.83	4.1566%
SRR2954155	PRJNA300310	SAMN04218941	coast	marsh	sediment	sediment metagenome	coastal sediment	29.2801	-89.97583	3.4662%
SRR5478611	PRJNA384096	SAMN06828347	ENVO:01000022	ENVO:01001305	ENVO:00002243		soil	29.44105	-89.93337	3.2073%
SRR1133196	PRJNA232457	SAMN02586135	coastal marsh		sediment		coastal sediment	29.45437	-89.77008	2.9762%
SRR5478617	PRJNA384096	SAMN06828341	ENVO:01000022	ENVO:01001305	ENVO:00002243		soil	29.44105	-89.93337	2.7445%
SRR6457335	PRJNA429259	SAMN08327666			seawater		marine water	28.5	-87.51	2.5993%
SRR6206202	PRJNA415515	SAMN07828062			wastewater treatment sludge	wastewater/sludge	wastewater/sludge	34.27	108.54	2.5198%
SRR5478587	PRJNA384096	SAMN06828371	ENVO:01000022	ENVO:01001305	ENVO:00002243		soil	29.44105	-89.93337	2.2916%
SRR5022896	PRJNA330773	SAMN05435905	-	-	-		freshwater	-32.06	115.83	2.0651%
SRR5469006	PRJNA381927	SAMN06820477	ENVO:01000022	ENVO:01001305	ENVO:00002243		soil	29.4406	-89.88492	1.7964%

SRR8505883	PRJNA510520	SAMN10797396			sediment		marine sediment	-75.003	165.012	1.6280%
SRR5478647	PRJNA384096	SAMN06828311	ENVO:01000022	ENVO:01001305	ENVO:00002243		soil	29.44105	-89.93337	1.6006%
SRR1948031	PRJNA279913	SAMN03449762	bohai sea	marine sediment	original soil sample added pyrene		marine sediment	37.6	120.35	1.4995%
SRR3156908	PRJNA310143	SAMN04481069	-	-	sediment		coastal sediment	6.542195	3.520752	1.4590%
SRR2953888	PRJNA300310	SAMN04218727	coast	marsh	sediment	sediment metagenome	coastal sediment	29.30231	-90.59848	1.3320%
SRR8609002	PRJNA523734	SAMN10986767	marine		Pyropia		host-associated	30.7	121.28	1.3058%
SRR7547252	PRJNA480575	SAMN09641961			Scrippsiella		host-associated	22.49	113.99	1.2779%
SRR3884070	PRJNA328594	SAMN05375666	marine	-	Water		marine water	19.45054	-120.0608	1.2688%
DRR061139	PRJDB4849	SAMD00051898	sediment	tsunami sediment	-		marine sediment	38.25	141.14	1.2439%
SRR2080568	PRJNA286014	SAMN03783534	Terrestrial	soil	soil		soil	18.32428	-65.8135	1.0699%
SRR5478593	PRJNA384096	SAMN06828365	ENVO:01000022	ENVO:01001305	ENVO:00002243		soil	29.44105	-89.93337	1.0556%
SRR8609022	PRJNA523734	SAMN10986748	marine		Pyropia		host-associated	30.7	121.28	1.0454%
SRR6517612	PRJNA431095	SAMN08384074	Wetland		sediment		coastal sediment	-32.9483	151.83472	1.0229%
SRR6457216	PRJNA429259	SAMN08327894			seawater		marine water	28.53	-87.5	0.9203%
SRR6457233	PRJNA429259	SAMN08327730			seawater		marine water	27	-89.99	0.8445%
SRR5860823	PRJNA395500	SAMN07407697	Wetland	Coastal wetland	Sediment		coastal sediment	22.49	114.03	0.8276%
SRR3156914	PRJNA310143	SAMN04481074	-	-	sediment		coastal sediment	6.542195	3.520752	0.7658%
SRR6457409	PRJNA429259	SAMN08327756			seawater		marine water	26.91	-89.07	0.7569%
SRR7547225	PRJNA480575	SAMN09641961			Scrippsiella		host-associated	22.49	113.99	0.7548%
SRR5478573	PRJNA384096	SAMN06828385	ENVO:01000022	ENVO:01001305	ENVO:00002243		soil	29.44105	-89.93337	0.7372%
SRR8609024	PRJNA523734	SAMN10986746	marine		Pyropia		host-associated	30.7	121.28	0.7244%
SRR5860820	PRJNA395500	SAMN07407698	Wetland	Coastal wetland	Sediment		coastal sediment	22.49	114.03	0.7051%

SRR5313221	PRJNA377204	SAMN06458653	lake	-	sediment		freshwater sediment	37.19	96.81	0.6783%
SRR1948037	PRJNA279913	SAMN03449763	bohai sea	marine sediment	original soil sample added pyrene		marine sediment	37.6	120.36	0.6781%
SRR3736205	PRJNA326707	SAMN05284610	estuarine biome		sediment	aquatic metagenome	coastal sediment	24.423	118.031	0.6431%
ERR713079	PRJEB8105	SAMEA3182252	wastewater treatment plant	wastewater treatment plant	activated sludge	wastewater/sludge	wastewater/sludge	55.95336	9.12846	0.6406%
SRR1566119	PRJNA260373	SAMN03020622	Terrestrial	land	soil	solid waste metagenome	soil	-3.4246	36.6796	0.6207%
SRR1562063	PRJNA259818	SAMN03014285	Solid waste	Soil	Soil		soil	-3.44	36.68	0.6202%
SRR5641666	PRJNA388861	SAMN07187879	Terrestrial		soil		soil	37.7	117.5	0.6188%
DRR061136	PRJDB4849	SAMD00051895	sediment	tsunami sediment	-		marine sediment	38.25	141.14	0.6134%
SRR3156814	PRJNA310143	SAMN04481075	-	-	sediment		coastal sediment	6.542195	3.520752	0.6116%
SRR5641664	PRJNA388861	SAMN07187869	Terrestrial		soil		soil	37.7	117.5	0.6108%
SRR5478618	PRJNA384096	SAMN06828340	ENVO:01000022	ENVO:01001305	ENVO:00002243		soil	29.44105	-89.93337	0.6097%
SRR3156904	PRJNA310143	SAMN04481068	-	-	sediment		coastal sediment	6.542195	3.520752	0.5929%
SRR2043654	PRJNA284669	SAMN03729528	lake	lake	sediment		hypersaline sediment	46.36	25.05	0.5870%
SRR6375411	PRJNA421083	SAMN08176556	marine	marine	Sediment		marine sediment	20.915898	70.362852	0.5852%
SRR6457229	PRJNA429259	SAMN08327565			seawater		marine water	28.04	-87.33	0.5793%
SRR5313233	PRJNA377204	SAMN06458641	lake	-	sediment		freshwater sediment	37.19	96.81	0.5613%
SRR6457506	PRJNA429259	SAMN08327768			seawater		marine water	26.99	-87.98	0.5583%
SRR8609031	PRJNA523734	SAMN10986755	marine		Pyropia		host-associated	30.7	121.28	0.5557%
SRR5860868	PRJNA395500	SAMN07407721	Wetland	Coastal wetland	Sediment		coastal sediment	22.49	114.03	0.5551%
SRR3156909	PRJNA310143	SAMN04481070	-	-	sediment		coastal sediment	6.542195	3.520752	0.5439%
SRR6396223	PRJNA421849	SAMN08200910			sediments		marine sediment	19.998578	110.60909	0.5406%
SRR5313245	PRJNA377204	SAMN06458629	lake	-	sediment		freshwater sediment	37.19	96.81	0.5342%

SRR3497608	PRJNA320997	SAMN04961898	-	-	soil		soil	43.5069	4.4547	0.5170%
SRR6457236	PRJNA429259	SAMN08327729			seawater		marine water	27	-89.99	0.4913%
SRR6435252	PRJNA428033	SAMN08280932			sediment		coastal sediment	13.32222	100.26365	0.4868%
SRR6457310	PRJNA429259	SAMN08327844			seawater		marine water	27.51	-89.01	0.4816%
SRR6457112	PRJNA429259	SAMN08327643			seawater		marine water	27	-85.99	0.4708%
SRR6457237	PRJNA429259	SAMN08327728			seawater		marine water	27	-89.99	0.4666%
SRR8609016	PRJNA523734	SAMN10986762	marine		Pyropia		host-associated	30.7	121.28	0.4626%
SRR8609019	PRJNA523734	SAMN10986761	marine		Pyropia		host-associated	30.7	121.28	0.4578%
SRR6457551	PRJNA429259	SAMN08327740			seawater		marine water	26.98	-89.98	0.4526%
SRR8609023	PRJNA523734	SAMN10986749	marine		Pyropia		host-associated	30.7	121.28	0.4510%
SRR6457309	PRJNA429259	SAMN08327845			seawater		marine water	27.51	-89.01	0.4410%
SRR8359317	PRJNA510850	SAMN10617125			activated sludge		wastewater/sludge	36.070337	120.41761	0.4402%
ERR2597124	PRJEB26931	SAMEA4688722	human	stomach	gastric mucosa	host-associated	host-associated	39.54	116.16	0.4314%
SRR7084774	PRJNA449342	SAMN09002943			Petroleum-contaminated sediment		coastal sediment	29.29	-89.91	0.4290%
SRR2349961	PRJNA295127	SAMN04044562	brackish lagoon	Natural zone of ecological interest	microbial mat	microbial mat metagenome	microbial mat/biofilm	43.45257	5.230085	0.4220%
SRR5860898	PRJNA395500	SAMN07407728	Wetland	Coastal wetland	Sediment		coastal sediment	22.49	114.03	0.4125%
SRR3736135	PRJNA326707	SAMN05284547	estuarine biome		sediment	aquatic metagenome	coastal sediment	24.465	117.1	0.4032%
SRR6457234	PRJNA429259	SAMN08327727			seawater		marine water	27	-89.99	0.3897%
DRR061137	PRJDB4849	SAMD00051896	sediment	tsunami sediment	-		marine sediment	38.25	141.14	0.3847%
SRR6457105	PRJNA429259	SAMN08327809			seawater		marine water	26.95	-87.53	0.3802%
SRR8599968	PRJNA522679	SAMN10956164	marine		Pyropia		host-associated	35.61	119.34	0.3800%
SRR6457318	PRJNA429259	SAMN08327850			seawater		marine water	27.5	-89	0.3799%

SRR1980853	PRJNA281530	SAMN03491550	marine pelagic biome	marine water body	water		marine water	13.96	-116.57	0.3721%
SRR6457384	PRJNA429259	SAMN08327829			seawater		marine water	26.99	-89.49	0.3629%
SRR8284947	PRJNA508441	SAMN10522681			Coastal water		coastal water	22.5	114	0.3590%
ERR713217	PRJEB8105	SAMEA3182390	wastewater treatment plant	wastewater treatment plant	activated sludge	wastewater/sludge	wastewater/sludge	55.50727	12.14549	0.3558%
SRR6457311	PRJNA429259	SAMN08327843			seawater		marine water	27.51	-89.01	0.3538%
SRR6457122	PRJNA429259	SAMN08327512			seawater		marine water	28	-87.5	0.3530%
SRR1303761	PRJNA248779	SAMN02800856	oil well16	oil well	water		oil-related	30.4	112.7	0.3394%
SRR1303705	PRJNA248705	SAMN02800801	lake environment53	lake	sediment		freshwater sediment	37.5	95.3	0.3256%
ERR713108	PRJEB8105	SAMEA3182281	wastewater treatment plant	wastewater treatment plant	activated sludge	wastewater/sludge	wastewater/sludge	55.95336	9.12846	0.3204%
SRR8285008	PRJNA508441	SAMN10522646			Coastal water		coastal water	22.5	113.98	0.3154%
DRR028744	PRJDB3419	SAMD00024531	coastal biome	natural environment	water		coastal water	29.95	121.92	0.3104%
SRR5313239	PRJNA377204	SAMN06458635	lake	-	sediment		freshwater sediment	37.19	96.81	0.3056%
SRR1980854	PRJNA281530	SAMN03491551	marine pelagic biome	marine water body	water		marine water	13.96	-116.57	0.2930%
SRR2349870	PRJNA295127	SAMN04044562	brackish lagoon	Natural zone of ecological interest	microbial mat	microbial mat metagenome	microbial mat/biofilm	43.45257	5.230085	0.2855%
SRR2001836	PRJNA281978	SAMN03565305	activated sludge	activated sludge	activated sludge	wastewater/sludge	wastewater/sludge	39.92	116.46	0.2850%
SRR6457235	PRJNA429259	SAMN08327726			seawater		marine water	27	-89.99	0.2840%
SRR7084779	PRJNA449342	SAMN09002950			Petroleum-contaminated sediment		coastal sediment	29.29	-89.91	0.2781%
SRR6457218	PRJNA429259	SAMN08327896			seawater		marine water	28.53	-87.5	0.2746%
SRR6457078	PRJNA429259	SAMN08327739			seawater		marine water	26.98	-89.98	0.2596%
SRR6457499	PRJNA429259	SAMN08327841			seawater		marine water	27.51	-89.01	0.2567%
SRR6457424	PRJNA429259	SAMN08327878			seawater		marine water	28.01	-87.51	0.2543%
SRR6457147	PRJNA429259	SAMN08327559			seawater		marine water	28	-87	0.2467%

SRR7084776	PRJNA449342	SAMN09002894			Petroleum-contaminated sediment		coastal sediment	29.29	-89.91	0.2450%
SRR6435245	PRJNA428033	SAMN08280935			sediment		coastal sediment	13.32222	100.26365	0.2420%
SRR8599963	PRJNA522679	SAMN10956171	marine		Pyropia		host-associated	35.61	119.34	0.2407%
SRR1562059	PRJNA259818	SAMN03014285	Solid waste	Soil	Soil		soil	-3.44	36.68	0.2372%
SRR6435242	PRJNA428033	SAMN08280928			sediment		coastal sediment	13.32222	100.26365	0.2367%
SRR2961644	PRJNA304214	SAMN04296796	deciduous woodland	wetland	sediment	sediment metagenome	coastal sediment	37.766944	119.16889	0.2324%
SRR8609014	PRJNA523734	SAMN10986756	marine		Pyropia		host-associated	30.7	121.28	0.2316%
SRR5641663	PRJNA388861	SAMN07187871	Terrestrial		soil		soil	37.7	117.5	0.2226%
SRR6457217	PRJNA429259	SAMN08327895			seawater		marine water	28.53	-87.5	0.2184%
SRR8599911	PRJNA522679	SAMN10956221	marine		Pyropia		host-associated	35.61	119.34	0.2170%
SRR3156867	PRJNA310143	SAMN04481078	-	-	sediment		coastal sediment	6.542195	3.520752	0.2168%
SRR5313241	PRJNA377204	SAMN06458633	lake	-	sediment		freshwater sediment	37.19	96.81	0.2108%
SRR5171552	PRJNA360666	SAMN06214480	Terrestrial	-	soil		soil	20.25	85.55	0.2079%
SRR6457427	PRJNA429259	SAMN08327877			seawater		marine water	28.01	-87.51	0.2065%
SRR6457548	PRJNA429259	SAMN08327864			seawater		marine water	27.51	-88	0.2041%
SRR8599967	PRJNA522679	SAMN10956165	marine		Pyropia		host-associated	35.61	119.34	0.2035%
SRR6457104	PRJNA429259	SAMN08327808			seawater		marine water	26.95	-87.53	0.2029%
SRR8599922	PRJNA522679	SAMN10956197	marine		Pyropia		host-associated	35.61	119.34	0.2029%
SRR8599918	PRJNA522679	SAMN10956193	marine		Pyropia		host-associated	35.61	119.34	0.1988%
SRR8599924	PRJNA522679	SAMN10956195	marine		Pyropia		host-associated	35.61	119.34	0.1975%
SRR5171520	PRJNA360666	SAMN06214480	Terrestrial	-	soil		soil	20.25	85.55	0.1967%
SRR5313223	PRJNA377204	SAMN06458651	lake	-	sediment		freshwater sediment	37.19	96.81	0.1957%

SRR5528686	PRJNA385860	SAMN06919907			sediment		freshwater sediment	37.15	-78.17	0.1943%
SRR8599937	PRJNA522679	SAMN10956219	marine		Pyropia		host-associated	35.61	119.34	0.1937%
SRR8599904	PRJNA522679	SAMN10956397	marine		Pyropia		host-associated	35.61	119.34	0.1917%
SRR6457178	PRJNA429259	SAMN08327778			seawater		marine water	27	-86.99	0.1913%
SRR8285007	PRJNA508441	SAMN10522645			Coastal water		coastal water	22.5	114	0.1911%
SRR6457426	PRJNA429259	SAMN08327876			seawater		marine water	28.01	-87.51	0.1891%
SRR8599949	PRJNA522679	SAMN10956407	marine		Pyropia		host-associated	35.61	119.34	0.1888%
SRR8609026	PRJNA523734	SAMN10986752	marine		Pyropia		host-associated	30.7	121.28	0.1887%
SRR2349942	PRJNA295127	SAMN04044563	brackish lagoon	Natural zone of ecological interest	microbial mat	microbial mat metagenome	microbial mat/biofilm	43.45257	5.230085	0.1885%
SRR8599914	PRJNA522679	SAMN10956226	marine		Pyropia		host-associated	35.61	119.34	0.1882%
SRR6457270	PRJNA429259	SAMN08327824			seawater		marine water	26.99	-89.49	0.1879%
SRR6457447	PRJNA429259	SAMN08327654			seawater		marine water	28.5	-87.52	0.1867%
SRR6457107	PRJNA429259	SAMN08327807			seawater		marine water	26.95	-87.53	0.1840%
SRR8609027	PRJNA523734	SAMN10986753	marine		Pyropia		host-associated	30.7	121.28	0.1821%
SRR5460968	PRJNA383082	SAMN06759285		Houxi midstream	water		freshwater	24.66	118.03	0.1665%
SRR7084472	PRJNA449342	SAMN09002890			Petroleum-contaminated sediment		coastal sediment	29.29	-89.91	0.1657%
SRR6457408	PRJNA429259	SAMN08327757			seawater		marine water	26.91	-89.07	0.1627%
SRR6457283	PRJNA429259	SAMN08327830			seawater		marine water	26.99	-89.49	0.1625%
SRR1980868	PRJNA281530	SAMN03491567	marine pelagic biome	marine water body	water	seawater metagenome	marine water	13.85	-116.63	0.1440%
SRR2349960	PRJNA295127	SAMN04044561	brackish lagoon	Natural zone of ecological interest	microbial mat	microbial mat metagenome	microbial mat/biofilm	43.45257	5.230085	0.1378%
SRR6457509	PRJNA429259	SAMN08327767			seawater		marine water	26.99	-87.98	0.1375%
SRR6457209	PRJNA429259	SAMN08327465			seawater		marine water	28.01	-88	0.1357%

SRR6457407	PRJNA429259	SAMN08327758			seawater		marine water	26.91	-89.07	0.1272%
SRR1460610	PRJNA253405	SAMN02870531	Ocean	open water	water		marine water	27.3672	-90.5779	0.1241%
SRR6176455	PRJNA419719	SAMN07789137	marine	sea water	water		marine water	29.635	-93.642	0.1165%
SRR8284967	PRJNA508441	SAMN10522620			Coastal water		coastal water	22.59	113.98	0.1128%
SRR6457319	PRJNA429259	SAMN08327840			seawater		marine water	27.51	-89.01	0.1115%
SRR6457329	PRJNA429259	SAMN08327791			seawater		marine water	27.01	-87.15	0.1095%
SRR6457212	PRJNA429259	SAMN08327900			seawater		marine water	29.01	-87.49	0.1090%
SRR7547240	PRJNA480575	SAMN09641961			Scrippsiella		host-associated	22.49	113.99	0.1077%
ERR1552122	PRJEB14970	SAMEA4359410	urban biome	research facility	microbial mat	microbial mat/biofilm	microbial mat/biofilm	48.973062	-119.4769	0.1054%
SRR3111419	PRJNA309048	SAMN04420617	-	-	Seawater	marine metagenome	Marine water	42.566025	34.183592	0.1051%
SRR3156903	PRJNA310143	SAMN04481067	-	-	sediment		coastal sediment	6.542195	3.520752	0.1021%
SRR3156815	PRJNA310143	SAMN04481076	-	-	sediment		coastal sediment	6.542195	3.520752	0.1017%
SRR8285009	PRJNA508441	SAMN10522647			Coastal water		coastal water	22.51	113.96	0.0988%
SRR3156846	PRJNA310143	SAMN04481077	-	-	sediment		coastal sediment	6.542195	3.520752	0.0979%
SRR5660915	PRJNA388981	SAMN07191707	river		sediment		freshwater sediment	38	117	0.0974%
ERR1942889	PRJEB20502	SAMEA103984233	estuarine biome	brackish estuary	estuarine water	water	marine water	-12.51	130.94	0.0970%
SRR3156813	PRJNA310143	SAMN04481066	-	-	sediment		coastal sediment	6.542195	3.520752	0.0893%
SRR2349845	PRJNA295127	SAMN04044561	brackish lagoon	Natural zone of ecological interest	microbial mat	microbial mat metagenome	microbial mat/biofilm	43.45257	5.230085	0.0846%
SRR6457317	PRJNA429259	SAMN08327851			seawater		marine water	27.5	-89	0.0844%
ERR2191484	PRJEB23334	SAMEA104368986	marine biome (ENVO:00000447)	oceanic bathypelagic zone biome (ENVO:01000037)	saline water (ENVO:00002010)	miscellaneous natural or artificial environment	marine water	40.641944	2.8505556	0.0815%
SRR6176441	PRJNA419719	SAMN07789071	marine	sea water	water		marine water	29.635	-93.642	0.0757%
SRR7084777	PRJNA449342	SAMN09002942			Petroleum-contaminated sediment		coastal sediment	29.29	-89.91	0.0752%

SRR8285010	PRJNA508441	SAMN10522648			Coastal water		coastal water	22.5	113.97	0.0716%
SRR6457327	PRJNA429259	SAMN08327789			seawater		marine water	27.01	-87.15	0.0677%
SRR6457159	PRJNA429259	SAMN08327854			seawater		marine water	27.5	-89	0.0674%
SRR8284945	PRJNA508441	SAMN10522679			Coastal water		coastal water	22.53	113.95	0.0619%
SRR6188401	PRJNA414763	SAMN07809461	coastal seawater	ocean	seawater		marine water	-5.99	-35.08	0.0589%
SRR6176463	PRJNA419719	SAMN07789072	marine	sea water	water		marine water	29.635	-93.642	0.0572%
SRR8284941	PRJNA508441	SAMN10522683			Coastal water		coastal water	22.51	113.96	0.0551%
SRR5861444	PRJNA395513	SAMN07409462	lake			aquatic metagenome	freshwater	46.3615	25.0509	0.0501%
SRR8284946	PRJNA508441	SAMN10522680			Coastal water		coastal water	22.5	114.01	0.0444%
SRR8284948	PRJNA508441	SAMN10522682			Coastal water		coastal water	22.5	113.98	0.0430%
SRR8371922	PRJNA511934	SAMN10651608	human gut	stool	stool		host-associated	17.424	78.4275	0.0329%
ERR713050	PRJEB8105	SAMEA3182223	wastewater treatment plant	wastewater treatment plant	activated sludge	wastewater/sludge	wastewater/sludge	56.65678	8.20202	0.0311%
SRR2954073	PRJNA300310	SAMN04218760	coast	marsh	sediment	sediment metagenome	coastal sediment	29.2801	-89.97583	0.0266%
SRR8371915	PRJNA511934	SAMN10651605	human gut	stool	stool		host-associated	17.424	78.4275	0.0262%
SRR8371934	PRJNA511934	SAMN10651594	human gut	stool	stool		host-associated	17.424	78.4275	0.0162%
SRR2953985	PRJNA300310	SAMN04218880	coast	marsh	sediment	sediment metagenome	coastal sediment	29.30231	-90.59848	0.0155%
SRR2954201	PRJNA300310	SAMN04218987	coast	marsh	sediment	sediment metagenome	coastal sediment	29.30231	-90.59848	0.0144%
SRR8241213	PRJNA506852	SAMN10475025	Terrestrial		soil	soil metagenome	soil	32.38	120.54	0.0134%
SRR2953925	PRJNA300310	SAMN04218820	coast	marsh	sediment	sediment metagenome	coastal sediment	29.2801	-89.97583	0.0091%
SRR5313229	PRJNA377204	SAMN06458645	lake	-	sediment	aquatic metagenome	freshwater sediment	37.19	96.81	0.0091%
SRR2953928	PRJNA300310	SAMN04218823	coast	marsh	sediment	sediment metagenome	coastal sediment	29.2801	-89.97583	0.0089%
SRR2954205	PRJNA300310	SAMN04218991	coast	marsh	sediment	sediment metagenome	coastal sediment	29.30231	-90.59848	0.0078%

SRR5641659	PRJNA388861	SAMN07187868	Terrestrial		soil		soil	37.7	117.5	0.0068%
SRR2954206	PRJNA300310	SAMN04218992	coast	marsh	sediment	sediment metagenome	coastal sediment	29.30231	-90.59848	0.0068%
SRR2953984	PRJNA300310	SAMN04218879	coast	marsh	sediment	sediment metagenome	coastal sediment	29.30231	-90.59848	0.0064%
SRR5478605	PRJNA384096	SAMN06828353	ENVO:01000022	ENVO:01001305	ENVO:00002243		soil	29.44105	-89.93337	0.0060%
SRR5860941	PRJNA395500	SAMN07407707	Wetland	Coastal wetland	Sediment		coastal sediment	22.49	114.03	0.0059%
ERR580987	PRJEB7017	SAMEA2707859	hypersaline	lake	sediment	hypersaline lake metagenome	hypersaline sediment	32.84455	119.3978	0.0057%
ERR1942875	PRJEB20502	SAMEA103984219	estuarine biome	estuarine mud	sediment	sediment metagenome	coastal sediment	-12.51	130.94	0.0056%
SRR5178036	PRJNA361424	SAMN06233428	marine	-	sediment	marine metagenome	marine sediment	22.44	72.13	0.0052%
SRR2136643	PRJNA291553	SAMN03945400	wastewater treatment plant	wastewater treatment plant	sludge	bioreactor metagenome	wastewater/sludge	45.76	126.69	0.0049%
SRR5860854	PRJNA395500	SAMN07407754	Wetland	Coastal wetland	Sediment		coastal sediment	22.49	114.03	0.0048%
SRR1291083	PRJNA214261	SAMN02786282	estuarine biome	estuarine sediment	sediment	sediment metagenome	coastal sediment	40.878	121.563	0.0047%
SRR1566041	PRJNA260373	SAMN03020622	Terrestrial	land	soil	solid waste metagenome	soil	-3.4246	36.6796	0.0046%
DRR061135	PRJDB4849	SAMD00051894	sediment	tsunami sediment	-	sediment metagenome	marine sediment	38.25	141.14	0.0045%
SRR5478607	PRJNA384096	SAMN06828351	ENVO:01000022	ENVO:01001305	ENVO:00002243		soil	29.44105	-89.93337	0.0045%
SRR5478649	PRJNA384096	SAMN06828309	ENVO:01000022	ENVO:01001305	ENVO:00002243		soil	29.44105	-89.93337	0.0044%
DRR061171	PRJDB4850	SAMD00051938	sediment	tsunami sediment	-	sediment metagenome	marine sediment	38.25	141.14	0.0043%
SRR5313235	PRJNA377204	SAMN06458639	lake	-	sediment	aquatic metagenome	freshwater sediment	37.19	96.81	0.0039%
SRR6457514	PRJNA429259	SAMN08327770			seawater		marine water	26.99	-87.98	0.0039%
DRR061167	PRJDB4850	SAMD00051933	sediment	tsunami sediment	-	sediment metagenome	marine sediment	38.25	141.14	0.0038%
SRR1303665	PRJNA248705	SAMN02800760	lake	lake	sediment	sediment metagenome	freshwater sediment	37.2	96.8	0.0037%
SRR2004058	PRJNA236538	SAMN03481568	soil	soil	soil	soil metagenome	soil	39.99	116.33	0.0035%
SRR5860828	PRJNA395500	SAMN07407739	Wetland	Coastal wetland	Sediment		coastal sediment	22.49	114.03	0.0033%

SRR5478608	PRJNA384096	SAMN06828350	ENVO:01000022	ENVO:01001305	ENVO:00002243		soil	29.44105	-89.93337	0.0033%
SRR5178043	PRJNA361424	SAMN06233421	marine	-	sediment	marine metagenome	marine sediment	21.07	72.07	0.0032%
SRR5478596	PRJNA384096	SAMN06828362	ENVO:01000022	ENVO:01001305	ENVO:00002243		soil	29.44105	-89.93337	0.0031%
ERR2264808	PRJEB24590	SAMEA104542027	blood plasma	blood plasma	blood plasma	host-associated	host-associated	39.9	116.3	0.0030%
SRR5478578	PRJNA384096	SAMN06828380	ENVO:01000022	ENVO:01001305	ENVO:00002243		soil	29.44105	-89.93337	0.0029%
SRR1566111	PRJNA260373	SAMN03020622	Terrestrial	land	soil	solid waste metagenome	soil	-3.4246	36.6796	0.0028%
ERR1942699	PRJEB20502	SAMEA103983947	estuarine biome	estuarine mud	sediment	sediment metagenome	coastal sediment	-12.51	130.94	0.0024%
SRR3984649	PRJNA328605	SAMN05357684	mangrove	-	soil	soil metagenome	soil	-23.01577	-43.36311	0.0024%
SRR3736123	PRJNA326707	SAMN05284540	estuarine biome		sediment	aquatic metagenome	coastal sediment	24.431	117.85	0.0023%
SRR5313237	PRJNA377204	SAMN06458637	lake	-	sediment	aquatic metagenome	freshwater sediment	37.19	96.81	0.0022%
DRR061188	PRJDB4850	SAMD00051945	sediment	tsunami sediment	-	sediment metagenome	marine sediment	38.25	141.14	0.0022%
SRR3736100	PRJNA326707	SAMN05284529	estuarine biome		sediment	aquatic metagenome	coastal sediment	24.465	117.8	0.0021%
DRR061145	PRJDB4849	SAMD00051904	sediment	tsunami sediment	-	sediment metagenome	marine sediment	38.25	141.14	0.0021%
SRR5320730	PRJNA378479	SAMN06546956	Terrestrial	marsh	soil	soil metagenome	soil	37.221464	-6.962177	0.0020%
SRR3736140	PRJNA326707	SAMN05284551	estuarine biome		sediment	aquatic metagenome	coastal sediment	24.423	118.025	0.0020%
SRR3736147	PRJNA326707	SAMN05284531	estuarine biome		sediment	aquatic metagenome	coastal sediment	24.431	117.849	0.0019%
SRR1923179	PRJNA236538	SAMN03417995	soil	soil	soil	soil metagenome	soil	39.99	116.33	0.0019%
SRR8552943	PRJNA521586	SAMN10906667	sludge2	bioreactor sludge	wastewater	bioreactor sludge metagenome	wastewater/sludge	41.130036	24.88649	0.0018%
DRR061140	PRJDB4849	SAMD00051899	sediment	tsunami sediment	-	sediment metagenome	marine sediment	38.25	141.14	0.0018%
SRR4527248	PRJNA351282	SAMN05952722	mangrove		sediment	soil metagenome	coastal sediment	22.4833	113.9833	0.0016%
DRR061176	PRJDB4850	SAMD00051946	sediment	tsunami sediment	-	sediment metagenome	marine sediment	38.25	141.14	0.0014%
DRR061173	PRJDB4850	SAMD00051941	sediment	tsunami sediment	-	sediment metagenome	marine sediment	38.25	141.14	0.0014%

DRR061180	PRJDB4850	SAMD00051912	sediment	tsunami sediment	-	sediment metagenome	marine sediment	38.25	141.14	0.0014%
DRR061169	PRJDB4850	SAMD00051935	sediment	tsunami sediment	-	sediment metagenome	marine sediment	38.25	141.14	0.0013%
DRR061168	PRJDB4850	SAMD00051934	sediment	tsunami sediment	-	sediment metagenome	marine sediment	38.25	141.14	0.0012%
DRR061154	PRJDB4850	SAMD00051914	sediment	tsunami sediment	-	sediment metagenome	marine sediment	38.25	141.14	0.0012%
SRR8552941	PRJNA521586	SAMN10906668	sludge3	bioreactor sludge	wastewater	bioreactor sludge metagenome	wastewater/sludge	41.130036	24.88649	0.0012%
SRR1560360	PRJNA259284	SAMN03009814	river sludge	river banks	water	freshwater sediment metagenome	freshwater sediment	-3.424616	36.6795	0.0012%
SRR1980861	PRJNA281530	SAMN03491558	marine pelagic biome	marine water body	water	seawater metagenome	marine water	13.88	-116.47	0.0012%
SRR5313225	PRJNA377204	SAMN06458649	lake	-	sediment	aquatic metagenome	freshwater sediment	37.19	96.81	0.0012%
DRR061179	PRJDB4850	SAMD00051950	sediment	tsunami sediment	-	sediment metagenome	marine sediment	38.25	141.14	0.0011%
SRR1980864	PRJNA281530	SAMN03491561	marine pelagic biome	marine water body	water	seawater metagenome	marine water	13.88	-116.47	0.0011%
SRR6457431	PRJNA429259	SAMN08327873			seawater		marine water	28.01	-87.51	0.0011%
ERR1552102	PRJEB14970	SAMEA4359390	urban biome	research facility	microbial mat	microbial mat/biofilm	microbial mat/biofilm	48.973062	-119.4769	0.0010%
SRR7547211	PRJNA480575	SAMN09641961			Scrippsiella		host-associated	22.49	113.99	0.0010%
SRR6457232	PRJNA429259	SAMN08327731			seawater		marine water	27	-89.99	0.0009%
SRR5313202	PRJNA377204	SAMN06458672	lake	-	sediment	aquatic metagenome	freshwater sediment	37.45	95.41	0.0009%
SRR5313220	PRJNA377204	SAMN06458654	lake	-	sediment	aquatic metagenome	freshwater sediment	37.19	96.81	0.0009%
SRR6428887	PRJNA427280	SAMN08226088	sponge tissue		tissue	sponge metagenome	host-associated	-23.00148	-42.55074	0.0009%
ERR1552277	PRJEB14970	SAMEA4359565	urban biome	research facility	microbial mat	microbial mat/biofilm	microbial mat/biofilm	48.973062	-119.4769	0.0009%
SRR2349956	PRJNA295127	SAMN04044565	brackish lagoon	Natural zone of ecological interest	microbial mat	microbial mat metagenome	microbial mat/biofilm	43.45257	5.230085	0.0009%
ERR1552283	PRJEB14970	SAMEA4359571	urban biome	research facility	microbial mat	microbial mat/biofilm	microbial mat/biofilm	48.973062	-119.4769	0.0008%
SRR5661020	PRJNA388981	SAMN07191707	river		sediment		freshwater sediment	38	117	0.0006%
SRR2961643	PRJNA304211	SAMN04296793	deciduous woodland	wetland	sediment	sediment metagenome	coastal sediment	37.76694	119.16888	0.0005%

SRR2961642	PRJNA304209	SAMN04296782	deciduous woodland	wetland	soil	soil metagenome	soil	37.76694	119.16888	0.0005%
SRR1462395	PRJNA253671	SAMN02886300	abalone seed nursing pond	abalone seed nursing pond	water	marine metagenome	marine water	22.47	15.21	0.0005%
SRR6987373	PRJNA427223	SAMN08225636	lake		water	freshwater metagenome	freshwater	39.0367	-84.1381	0.0005%
SRR2579284	PRJNA298100	SAMN04155558	Terrestrial	soil	soil	soil metagenome	soil	39.95	116.28	0.0003%
SRR5262288	PRJNA374976	SAMN06341004	Terrestrial	-	soil	soil metagenome	soil	18.73	110.2	0.0003%
SRR6304518	PRJNA358048	SAMN06112733	lake	-	water	aquatic metagenome	freshwater	31.53	120.21	0.0001%
SRR5666078	PRJNA390005	SAMN07212121	seashore	pond	water	gut metagenome	host-associated	21.68	110.88	0.0001%

Table S6. Functional annotation of FT118-specific orthogroups.

Orthogroup	EGGNOG Annotation	#genes
OG0000044	COG0643 Chemotaxis protein histidine kinase and related kinases	1
	His Kinase A (phosphoacceptor) domain	5
	Histidine kinase	1
	PAS domain	1
	PAS fold	1
	PhoQ Sensor	7
	Response regulator receiver	1
OG0000672	ATP-binding region ATPase domain protein	1
	Helix-turn-helix domain	1
	Helix-turn-helix XRE-family like proteins	1
	His Kinase A (phosphoacceptor) domain	4
	Histidine kinase	1
	transcriptional regulator	1
OG0001123	Belongs to the sigma-70 factor family. ECF subfamily	2
	Histidine kinase	1
	In Caulobacter crescentus, CC3477 is differentially expressed in minimal salts media with glucose as compared to complex media	1
	Sigma-70 region 2	1
	Sigma-70, region 4	3
OG0001410	Hydantoinase/oxoprolinase	2
	Hydantoinase/oxoprolinase N-terminal region	2
	N-methylhydantoinase A acetone carboxylase, beta subunit	3
OG0001421	diguanylate cyclase	2
	PFAM EAL domain	1
	Putative diguanylate phosphodiesterase	4
OG0001428	Belongs to the binding-protein-dependent transport system permease family	7
OG0001430	Bacterial regulatory proteins, tetR family	4

	transcriptional regulator	3
OG0001626	PFAM TadE family protein	2
	Putative Flp pilus-assembly TadE/G-like	4
OG0001627	Pyruvate 2-oxoglutarate dehydrogenase complex dehydrogenase (E1) component eukaryotic type beta subunit	2
	Transketolase, pyrimidine binding domain	4
OG0001774	COG1522 Transcriptional regulators	1
	helix_turn_helix ASNC type	3
	transcriptional	1
OG0001785	Bacterial periplasmic substrate-binding proteins	1
	Binding-protein-dependent transport system inner membrane component	4
OG0001869	Filamentation induced by cAMP protein fic	1
OG0001878	ATPase activator activity	1
	DnaJ molecular chaperone homology domain	3
OG0001888	Adenylate and Guanylate cyclase catalytic domain	1
	Adenylate cyclase	2
	Transcriptional regulatory protein, C terminal	1
OG0001889	COG0665 Glycine D-amino acid oxidases (deaminating)	1
	FAD dependent oxidoreductase	2
	Sarcosine oxidase beta subunit	1
OG0001890	COG0589 Universal stress protein UspA and related nucleotide-binding proteins	1
	Universal stress protein	1
	Universal stress protein family	2
OG0001899	ABC-type nitrate sulfonate bicarbonate transport systems, periplasmic components	1
	COG0715 ABC-type nitrate sulfonate bicarbonate transport systems periplasmic components	1
	NMT1-like family	2
OG0001910	unannotated	4
OG0001911	unannotated	4
OG0001982	Flp Fap pilin component	2
OG0001987	DUF35 OB-fold domain, acyl-CoA-associated	2

	Rubredoxin-like zinc ribbon domain (DUF35_N)	1
OG0001991	Glycosyl transferase family group 2	1
	transferase activity, transferring glycosyl groups	1
	Type II secretion system (T2SS), protein E, N-terminal domain	1
OG0001993	Chorismate mutase type II	1
OG0001997	Chain length determinant family protein	1
	Chain length determinant protein	1
	Involved in chromosome partitioning	1
OG0002007	Nitrile hydratase beta subunit	2
OG0002009	unannotated	3
OG0002015	2Fe-2S iron-sulfur cluster binding domain	1
	Belongs to the GcvT family	2
OG0002025	PAS domain	3
OG0002035	Major facilitator Superfamily	3
OG0002136	Phosphoadenosine phosphosulfate reductase family	1
	Reduction of activated sulfate into sulfite	1
OG0002137	Asp Glu Hydantoin racemase family protein	1
	Maleate cis-trans isomerase	1
OG0002140	AsmA family	1
	protein involved in outer membrane biogenesis	1
OG0002143	FliM is one of three proteins (FliG, FliN, FliM) that forms the rotor-mounted switch complex (C ring), located at the base of the basal body. This complex interacts with the CheY and CheZ chemotaxis proteins, in addition to contacting components of the motor that determine the direction of flagellar rotation	2
OG0002145	ATP synthase B/B' CF(0)	1
	Component of the F(0) channel, it forms part of the peripheral stalk, linking F(1) to F(0)	1
OG0002146	ATP synthase subunit C	1
	F(1)F(0) ATP synthase produces ATP from ADP in the presence of a proton or sodium gradient. F-type ATPases consist of two structural domains, F(1) containing the extramembraneous catalytic core and F(0) containing the membrane proton channel, linked together by a central stalk and a peripheral stalk. During catalysis, ATP synthesis in the catalytic domain of F(1) is coupled via a rotary mechanism of the central stalk subunits to proton translocation	1
OG0002147	it plays a direct role in the translocation of protons across the membrane	2

OG0002148	Putative F0F1-ATPase subunit Ca2+/Mg2+ transporter	2
OG0002149	Produces ATP from ADP in the presence of a proton gradient across the membrane	2
OG0002150	Protein of unknown function (DUF2892)	1
	Rhodanese Homology Domain	1
OG0002152	PEP-CTERM motif	1
OG0002155	Glyoxalase-like domain	2
OG0002160	Putative beta-barrel porin 2	2
OG0002162	Glycosyl transferases group 1	1
	glycosyltransferase	1
OG0002163	Sulfotransferase domain	1
	Sulfotransferase family	1
OG0002172	unannotated	2
OG0002174	unannotated	2
OG0002175	COG2771 DNA-binding HTH domain-containing proteins	1
	helix_turn_helix, Lux Regulon	1
OG0002176	Glycosyl transferase, family 2	1
	protein transport	1
OG0002178	unannotated	2
OG0002181	Endoribonuclease L-PSP	2
OG0002182	Divergent polysaccharide deacetylase	1
	protein conserved in bacteria	1
OG0002185	Protein of unknown function (DUF3485)	1
	Transmembrane exosortase (Exosortase_EpsH)	1
OG0002187	Sarcosine oxidase subunit delta	1
	Sarcosine oxidase, delta subunit	1
OG0002192	protein conserved in bacteria	2
OG0002193	unannotated	2
OG0002196	MotA/TolQ/ExbB proton channel family	1

	With MotB forms the ion channels that couple flagellar rotation to proton sodium motive force across the membrane and forms the stator elements of the rotary flagellar machine	1
OG0002197	chemotaxis MotB protein	1
	PFAM OmpA MotB domain protein	1
OG0002200	peptidylprolyl isomerase	2
OG0002204	Belongs to the WrbA family	1
	NADPH-dependent FMN reductase	1
OG0002205	unannotated	2
OG0002206	protein possibly involved in aromatic compounds catabolism	1
	protein, possibly involved in aromatic compounds catabolism	1
OG0002207	unannotated	2
OG0002210	AraC-like ligand binding domain	1
	Cupin	1
OG0002218	von Willebrand factor (vWF) type A domain	2
OG0002228	Glycosyltransferase family 28 C-terminal domain	2
OG0002232	COG3000 Sterol desaturase	1
	Fatty acid hydroxylase superfamily	1
OG0002234	unannotated	2
OG0002241	Acetyl-CoA hydrolase/transferase C-terminal domain	1
	PFAM Acetyl-CoA hydrolase transferase	1
OG0002247	Type II secretion system (T2SS), protein F	2
OG0002248	COG1226 Kef-type K transport systems	1
	Ion channel	1
OG0002249	Dodecin	2
OG0002256	Predicted permease	2
OG0002259	PFAM thioesterase superfamily	1
	Thioesterase superfamily	1
OG0002266	COG1840 ABC-type Fe3 transport system, periplasmic component	1
	Required for the activity of the bacterial periplasmic transport system of putrescine	1

OG0002270	FAD dependent oxidoreductase	2
OG0002280	Belongs to the pirin family	2
OG0002284	unannotated	2
OG0002287	CrcB-like protein, Camphor Resistance (CrcB)	1
	Important for reducing fluoride concentration in the cell, thus reducing its toxicity	1

Table S7. Measured environmental variables of mangrove sediment samples.

Site	Depth (cm)	Temperature (°C)	pH	Salinity (‰)	NH4+ (mg/g)	NO3- (ug/g)	NO2- (ug/g)	Moisture	Dry/wet	TN (mg/g)	TON	IN/TN	TC (mg/g)	TOC (mg/g)	TP (mg/g)	TOC/TON
MF1	0-2	38	7.47	4	0.0120	0.2573	0.0774	0.5782	0.4218	0.5913	0.5790	0.0209	7.4328	6.8723	0.5098	13.8478
MF1	6-8	36	7.42	4	0.0198	0.2470	0.0557	0.6243	0.3757	0.6352	0.6152	0.0316	6.4181	6.3614	0.3834	12.0646
MF1	12-14	33	7.85	4	0.0258	0.0776	0.0891	0.5016	0.4984	0.7365	0.7105	0.0352	11.4264	10.4241	0.5236	17.1161
MF1	20-22	30	8.15	5	0.0234	0.7257	0.0586	0.4474	0.5526	0.6313	0.6072	0.0382	8.1854	7.8613	0.4466	15.1045
MF1	28-30	28	8.25	6	0.0086	0.7716	0.0701	0.2724	0.7276	0.8235	0.8140	0.0115	10.8697	9.9818	0.7311	14.3067
MG1	0-2	30	7.49	4	0.0200	0.3155	0.0615	0.4796	0.5204	0.7893	0.7689	0.0259	9.0442	8.5605	0.6060	12.9899
MG1	6-8	29.5	7.68	4	0.0318	1.0319	0.1007	0.5501	0.4499	0.6711	0.6382	0.0491	8.9258	8.8946	0.5254	16.2609
MG1	12-14	29	8.01	5	0.0337	0.6683	0.0838	0.5111	0.4889	0.6137	0.5793	0.0561	8.0053	7.8389	0.5276	15.7874
MG1	20-22	28.5	8.09	6	0.0248	1.4665	0.0506	0.4846	0.5154	0.5917	0.5653	0.0445	7.7151	7.4796	0.4073	15.4350
MG1	28-30	28	8.09	10	0.0245	1.3025	0.1169	0.4845	0.5155	0.3969	0.3710	0.0652	6.1692	5.6992	0.4510	17.9207

References

(1) Peterson BG, Carl P (2020). _PerformanceAnalytics: Econometric Tools for Performance and Risk Analysis_. R package version 2.0.4, <https://CRAN.R-project.org/package=PerformanceAnalytics>.

(2) LEIFSON E. The Bacterial Flora of Distilled And Stored Water. II. Caulobacter vibrioides Henrici and Johnson 1935 in distilled water. Int J Syst Evol Microbiol. 1962;12(4):155-60.

(3) Sun LN, Yang ED, Wei JC, Tang XY, Cao YY, Han GM. Caulobacter flavus sp. nov., a stalked bacterium isolated from rhizosphere soil. Int J Syst Evol Microbiol. 2015;65(12):4374-80.

(4) Cho JC, Giovannoni SJ. Parvularcula bermudensis gen. nov., sp. nov., a marine bacterium that forms a deep branch in the alpha-Proteobacteria. Int J Syst Evol Microbiol. 2003;53(Pt 4):1031-6.

(5) Harbison AB, Price LE, Flythe MD, Bräuer SL. Micropepsis pineolensis gen. nov., sp. nov., a mildly acidophilic alphaproteobacterium isolated from a poor fen, and proposal of Micropepsaceae fam. nov. within Micropepsales ord. nov. Int J Syst Evol Microbiol. 2017;67(4):839-44.

(6) Tully BJ, Graham ED, Heidelberg JF. The reconstruction of 2,631 draft metagenome-assembled genomes from the global oceans. Scientific Data. 2018;5:170203.

(7) Parks DH, Rinke C, Chuvochina M, Chaumeil P-A, Woodcroft BJ, Evans PN, et al. Recovery of nearly 8,000 metagenome-assembled genomes substantially expands the tree of life. Nature Microbiology. 2017;2(11):1533-42.
